# Leveraging Multiple Data Streams for Prioritization of Mixtures for Hazard Characterization

**DOI:** 10.3390/toxics10110651

**Published:** 2022-10-29

**Authors:** Brianna N. Rivera, Christine C. Ghetu, Yvonne Chang, Lisa Truong, Robyn L. Tanguay, Kim A. Anderson, Susan C. Tilton

**Affiliations:** Department of Environmental and Molecular Toxicology, Oregon State University, Corvallis, OR 97331, USA

**Keywords:** mixtures, chemical prioritization, polycyclic aromatic hydrocarbons, sufficiently similar mixtures, mixtures safety assessment

## Abstract

There is a growing need to establish alternative approaches for mixture safety assessment of polycyclic aromatic hydrocarbons (PAHs). Due to limitations with current component-based approaches, and the lack of established methods for using whole mixtures, a promising alternative is to use sufficiently similar mixtures; although, an established framework is lacking. In this study, several approaches are explored to form sufficiently similar mixtures. Multiple data streams including environmental concentrations and empirically and predicted toxicity data for cancer and non-cancer endpoints were used to prioritize chemical components for mixture formations. Air samplers were analyzed for unsubstituted and alkylated PAHs. A synthetic mixture of identified PAHs was created (Creosote-Fire Mix). Existing toxicity values and chemical concentrations were incorporated to identify hazardous components in the Creosote-Fire Mix. Sufficiently similar mixtures of the Creosote-Fire Mix were formed based on (1) relative abundance; (2) toxicity values; and (3) a combination approach incorporating toxicity and abundance. Hazard characterization of these mixtures was performed using high-throughput screening in primary normal human bronchial epithelium (NHBE) and zebrafish. Differences in chemical composition and potency were observed between mixture formation approaches. The toxicity-based approach (Tox Mix) was the most potent mixture in both models. The combination approach (Weighted-Tox Mix) was determined to be the ideal approach due its ability to prioritize chemicals with high exposure and hazard potential.

## 1. Introduction

### 1.1. Complexity of Studying Environmental Mixtures

Toxicological studies have traditionally focused on studying one chemical at a time; however, people are exposed to complex mixtures of chemicals [1,2]. The US EPA, NIEHS, and NRC have recognized the need to assess chemical mixture exposures [3]. The composition and concentrations of environmental mixtures are constantly changing resulting in an unknown number of unique environmental mixtures [4,5]. For example, a group of just 20 different chemicals would result in more than 1 million possible chemical combinations. To simplify complex environmental mixtures for hazard characterization, approaches to prioritize chemicals in a mixture of interest have been identified. It is known that chemical combinations in an environmental mixture do not occur randomly [5]. Environmental factors that can be used to prioritize mixtures include source of exposure (i.e., emission source), exposure concentrations, environmental fate, exposure population, biomonitoring, and environmental medium (i.e., chemicals found in air) [6,7,8,9,10,11]. The potential for chemicals to cause biological effects has also been incorporated as a way to prioritize chemicals for hazard characterization. Criteria for effect-based prioritization include similar mechanism of action, bioactivity, specific toxicological effects, target organs or human health risk [1,6,12,13,14,15,16]. More recent approaches have incorporated both exposure and potential to cause biological effects; providing a more holistic perspective on understanding mixture toxicity [13,17].

#### 1.1.1. Component-Based Approach

Once a mixture of interest has been identified risk assessment of this mixture may be carried out using several different approaches. Mixtures risk assessment is most often carried out using a component-based approach due to the lack of toxicity information on the mixture of interest [18]. Component-based approaches evaluate single chemicals rather than mixtures [19,20]. This approach incorporates available toxicity values of individual components and assumes additivity or toxicological similarity [18,21]. Limitations of the component-based approach include lack of toxicity information for identified components and inherent assumptions of the type of interaction occurring between chemicals in the mixture, which may not adequately predict toxicity of the whole mixture [22,23,24].

#### 1.1.2. Whole Mixture Approach

Although component-based approaches are useful when no mixture toxicity data are available, mixtures-based approaches are preferred [6,25,26]. Current mixtures-based approaches begin with collection of dose–response data for the mixture of interest for toxicity value derivation. One of the most common mixtures-based approaches is the whole mixture approach. The whole mixture approach includes collecting and testing a whole environmental extract or fractionating the extract by physicochemical properties and testing in bioassays to identify which components of the mixture may be driving toxicity [27,28]. Limitations of the whole mixture or fractionation approach are that it can be expensive and laborious due to use of the whole sample extract and fractionation process [25,26]. This approach is also limited to chemicals that the researcher is able to be identify in the environmental sample, which could result in unidentified chemicals being the drivers of toxicity in the mixture. To account for this, toxicant confirmation procedures are typically applied. This may involve creation of the mixture of identified compounds for bioassay screening or use of in silico models to identify components of the toxic fraction [27,29].

#### 1.1.3. Sufficiently Similar or Representative Mixtures

The formation of sufficiently similar or representative mixtures is another mixture approach that may be used in tandem with the whole mixture approach described above or on its own. Different types of mixtures may be formed depending on the research question and selected prioritization approach. The U.S. EPA defines sufficiently similar mixtures as a mixture with chemical composition and proportions that are similar to an environmental mixture of interest [23]. A representative mixture can be defined as a subset of prioritized chemicals that are representative of relevant co-exposures or similar biological effects, but not associated with a single environmental mixture. Screening of representative mixtures may serve as a way to prioritize mixtures for further toxicological investigations, such as to investigate interactions between components within the mixture [30]. The sufficiently similar mixture approach uses toxicological data from a well-characterized, often simpler, mixture that can then be used to predict the toxicity of an untested mixture of interest [23,31].

In some cases, existing toxicity data for a mixture that is determined to be “sufficiently similar” to a mixture of interest may be used. In other cases, a sufficiently similar mixture may be created. To create sufficiently similar or representative mixtures, the components within the mixture must first be prioritized, however, composition may vary based on the prioritization approach [14,32]. Chemical components can be prioritized based on environmental factors, biological effects or a combination approach that incorporates exposure and biological effects [6,17,33,34]. New approach methodologies (NAMs) incorporating multiple data streams have been implemented for chemical prioritization. These data streams may include toxicity data from in vivo, in vitro, or in silico models and/or exposure information [13,16,17,35].

Specific methods regarding what is defined as sufficient similarity remain to be a topic of discussion within the mixture community. According to EPA guidance this is based on the chemical composition and proportions in the mixture. Questions exist as to which components need to overlap between the mixture of interest and the sufficiently similar mixture. Based on chemical prioritization approach used, composition of the sufficiently similar mixture may differ resulting in differences in biological effects. To account for this, comparison of chemical composition and toxicological data of the sufficiently similar mixture and mixture of interest have been implemented. Although it is often the case that toxicological data do not exist for the mixture of interest to make direct comparisons to; resulting in reliance on comparison of chemical composition to deem sufficient similarity.

### 1.2. Exposure to Polycyclic Aromatic Hydrocarbons and Associated Health Effects

Polycyclic aromatic hydrocarbons (PAHs) are a large class of organic compounds that consist of two or more fused rings [36,37,38]. They are a wide-spread environmental pollutant with natural or anthropogenic sources, and often exist as complex mixtures [39,40,41]. Anthropogenic sources include, but are not limited to biomass burning, incomplete fossil fuel combustion, oil spills, or industrial processes [42,43]. PAHs are found in petrochemicals or can be emitted through natural processes such as volcanic eruptions or forest fires [43,44,45]. In this study, our sampling site consisted of multiple sources of exposure, including creosote and wildfire smoke resulting in a real-world complex mixture of PAHs.

Humans can be exposed to PAHs through inhalation, ingestion or dermal exposure [36,46]. Individuals are primarily exposed through inhalation or ingestion [47,48,49]. PAHs are highly lipophilic compounds that easily pass through the biological membrane and are widely distributed in the body and are detectable in most human tissues [47]. Short-term exposures have been reported to have impacts on the lung function of asthmatics and thrombotic effects for individuals with coronary artery disease [36]. There have also been associations between PAH metabolites and reduced lung function in individuals without underlying health conditions [47,49,50]. Additionally, the lung has been reported as a major target organ for PAH carcinogenicity with an increased risk of lung cancer with inhalation exposure [48,51]. A number of PAHs have been reported as being mutagenic or genotoxic, and they have also been shown to impact reproductive and immune function [47,52,53]. Prenatal exposure to PAHs has been shown to have effects on birth outcomes and developmental effects such as neurodevelopmental or behavioral problems [54,55,56,57].

### 1.3. Study Objectives

Currently, there is no clear guidance regarding how to form sufficiently similar mixtures. To our knowledge, comparisons between exposure-based, effect-based, or combination approaches using the same environmental sample have not been explored. The first objective of this study is to compare mixture composition for exposure, effect and combination-based approaches. Incorporation of multiple data streams using real-world chemical concentrations and toxicity data from in vivo and in silico models was implemented for chemical prioritization. The use of multiple data streams supplemented toxicity data gaps for PAHs and captured the diversity of biological impacts of PAHs. Availability of toxicity data for PAHs and differences in chemical prioritization for traditional versus non-traditional toxicity metrics were also explored. The second objective of this study is to compare differences in bioactivity and potency based on mixture composition. Comparisons were made using in vitro human bronchial epithelium and in vivo early life-stage zebrafish. Incorporation of in vitro and in vivo model systems were utilized based on previously established methods for high-throughput chemical prioritization [30].

## 2. Materials and Methods

### 2.1. Objective 1: Chemical Prioritization and Mixture Formation

#### 2.1.1. Sample Site, Field Sampling, and Analysis

Environmental sampling occurred at a location in which wood preservation had historically taken place using coal tar creosote. While sampling in the summer of 2017, a wildfire broke out approximately 121 km away and got as close as 88.5 km, which had significant impacts on air quality in the region [58]. The impact of the nearby wildfire in conjunction with existing contamination at the sampling site resulted in a complex mixture of PAHs captured by stationary air samplers. Passive air sampling took place using air cages containing five low-density polyethylene strips [59]. Air samplers were placed at 5 separate locations along the shoreline of the site and deployed for 30 days [58]. One triplicate sampling site was also included for quality control purposes. Procedures used for conditioning, cleaning, sampling transport, and extraction, can be found elsewhere [59,60]. Sample extracts were concentrated down to 1 mL and spiked with an internal standard, perylene-d12. Sample aliquots were analyzed for 63 PAHs using an Agilent 7890B gas chromatograph with an Agilent 7000C triple quadrupole mass spectrometer (Table S1) [61]. Air concentrations were then calculated from instrumental concentrations as previously described [58,62]. Air concentrations for persistent organic pollutants, such as PAHs, are known to be fairly uniform across co-located sampling locations [63]. Therefore, an average of the estimated environmental concentrations for each detected compound from the five sampling locations was used for mixture formations (Table S2).

#### 2.1.2. Collection of Toxicity Metrics

Human health toxicity values for cancer and non-cancer endpoints were collected from federal and state databases (Table 1). Toxicity values were initially obtained from the Integrated Risk Information System (IRIS) database [64]. The U.S. Environmental Protection Agency’s software, Comptox Chemicals Dashboard was then used to obtain additional toxicity values [65]. Sources of toxicological information were prioritized based on the Environmental Protection Agency’s Office of Solid Waste and Emergency Response policy recommendations [66]. A quantitative structure activity relationship (QSAR) model called conditional toxicity value (CTV) was also used for chemicals lacking toxicity values [67]. Toxicity testing in zebrafish was conducted for the 63 PAHs that samples were analyzed for and benchmark concentrations values were calculated [68]. International Agency for Research on Cancer (IARC) cancer classification and different sources of potency including relative potency factor (RPF) were also incorporated [69]. RPF was obtained from the Integrated Risk Information System (IRIS) database and most conservative values were selected [64]. Toxic Equivalency Factor (TEF) was obtained from Samburova et al. which used RPF values from Nisbet and LaGoy to calculate values for 88 PAHs using structurally similar isomers [70,71] A full list of toxicity values for each PAH in this study can be found in Table S6.

#### 2.1.3. Chemical Prioritization

A synthetic mixture of all detected PAHs was first created. Three different mixture formation approaches were then further explored. An exposure-based approach selected chemicals strictly based on chemical abundance. This mixture selected the top seven most abundant PAHs. A toxicity-based (Toxicity Mix) and combination approach (weighted-toxicity mixture) were also explored. The toxicity-based approach incorporated chemical abundance, relative potency factors (RPF), cancer potency value (CPV), inhalation unit risk (IUR), reference concentration (RfC), oral slope factor (OSF), reference dose (RfD), and zebrafish benchmark concentration (BMC) to prioritize chemical components (Equation (1)). The weighted-toxicity mixture, which incorporated hazard and abundance, first calculated the proportion of each chemical in the simple PAH mixture using Equation (2). Toxicity metrics were then multiplied by the proportion of total to weight each metric by its chemical abundance using Equation (3). Toxicity metrics for weighted-toxicity mixture included toxic equivalency factors (TEF), inhalation unit risk (IUR), reference concentration (RfC), oral slope factor (OSF), reference dose (RfD), IARC classification, and zebrafish benchmark concentration (BMC) to prioritize chemical components. Toxicity metrics for both approaches were compiled into a spreadsheet with each toxicity metric in its own respective column. Chemicals were prioritized by sorting each weighted or unweighted toxicity metric from high to low hazard and given a rank 1 through 30, i.e., BMC values were sorted from low to high and the chemical with the lowest BMC value was given a ranking of 1. An average rank was then calculated by taking the average of the individual toxicity value rankings (Equation (1) or Equation (4)). The top seven ranking chemicals were then selected to be used in the sufficiently similar mixtures.

Toxicity mix chemical prioritization approach

(1) $$Average\;Rank\;Toxicity\;Mix=\frac{\begin{array}{c} Chemical\;Abundance\;Rank+RPF\;Rank+CPV\;Rank\\ +IUR\;Rank+RfC\;Rank\\ +OSF\;Rank+RfD\;Rank\\ +Zebrafish\;BMC50\;Rank \end{array}}{Total\;\#\;of\;Rankings}$$

Weighted-toxicity mix chemical prioritization approach

(2) $$Proportion\;of\;Total=\frac{Mass/Volume\;of\;Chemical}{Mass/Volume\;of\;Mixture}$$

Mass/Volume is equal to ng/m^3^.

(3) Weighted Toxicity Value=Proportion of Total ∗Toxicity Value

(4) $$Average\;Rank\;Weighted\;Toxicity\;Mix=\frac{\begin{array}{c} IARC\;Classification\;Rank+TEF\;Rank\\ +IUR\;Rank+RfC\;Rank\\ +OSF\;Rank+RfD\;Rank\\ +Zebrafish\;BMC50\;Rank \end{array}}{Total\;\#\;of\;Rankings}$$

#### 2.1.4. Correlation of Toxicity Metric Rankings

Correlations between toxicity value rankings for each chemical were compared in order to identify similarities in chemical rankings based on toxicity metric. Correlations between chemical rankings were investigated using “ggcorrplot” in R studio (version 4.1.1) [73,74]. Significant correlations were defined with a p-value cut-off of 0.05. Correlations were calculated using Pearson correlations for complete observations only. Correlation plots were generated using a correlation matrix and a matrix of correlation p-values. Pairs of chemicals were labeled with their correlation coefficients. Values with a p-value greater than 0.05 were denoted with an X.

#### 2.1.5. Chemical Mixture Preparation

Mixtures were created using environmental proportions from the average concentrations described above. Chemicals in the mixture were scaled relative to the most abundant chemical based on respective environmental concentrations. Neat chemical standards were purchased and dissolved in ACS grade n-hexane or toluene and stored at 4 °C until use (Thermo Fisher Scientific, Waltham, MA, USA). An aliquot of each mixture was taken and spiked with a known concentration of analytical surrogates in order to quantify concentrations of target analytes in the mixture. Concentrations were confirmed on an Agilent 7890B gas chromatograph with an Agilent 7000C triple quadrupole mass spectrometer using the same PAH analytical method for sample analysis [61]. Expected concentrations within 10% of reported concentrations met data quality objectives. Stock solutions for each mixture were solvent exchanged into ACS grade dimethyl sulfoxide (DMSO) and stored at room temperature (Thermo Fisher Scientific, Waltham, MA, USA). Table S3 details chemical name, CAS registry numbers, supplier information, and purity.

### 2.2. Objective 2: Comparison of Mixture Toxicity

#### 2.2.1. Normal Human Bronchial Epithelium Maintenance

Normal Human Bronchial Epithelial cells (NHBE) (passage 4; Lonza, Walkersville, MD, USA) were expanded in a culture flask in Pneumacult-Ex Plus media (STEMCELL Technologies, Vancouver, Canada) until 90% confluency was reached. Once confluency was reached, cells were washed with Dulbecco Phosphate Buffer Solution (Thermo Fisher Scientific, Waltham, MA, USA) trypsinized and seeded into black-walled 96-well plates at 1.8 × 10^4^ cells/well in 200 µL Pneumacult-Ex Plus media. Cells were maintained at 37 °C and 5% CO_2_ and media was changed every other day until 90% confluency was reached. Chemical stocks were diluted to 2% DMSO (*v*/*v*) with Pneumacult Ex media (STEMCELL Technologies, Vancouver, Canada). Cells were exposed for 24 h at 37 °C and 5% CO_2_. Initial range finding experiments were conducted with a seven-point dose response. Mixture concentrations were selected based on concentrations at which concentration-response curves could be generated and EC_50_ values could be calculated, or due to physical constraints such as limits of solubility.

##### Lactate Dehydrogenase Assay

Cytotoxicity was measured based on the release of lactate dehydrogenase (LDH) using the Cyquant Lactate Dehydrogenase Colorimetric Assay (Thermo Fischer Scientific, Waltham, MA, USA). After the exposure period was complete, an equal volume of cell media and LDH reagents were transferred to a clear 96-well plate and incubated away from light for 30 min. An equal volume of stop solution was then added, and absorbance was read at 490 nm and background was read at 680 nm using Synergy HTX plate Bio Tek plate reader (Winooski, VT, USA). Cytotoxicity was calculated by subtracting background from absorbance. Plates containing cells were then used for one of the remaining intracellular assays.

##### Cell Titer Glo Assay

Cell viability was measured based on quantification of ATP, which serves as an indicator of metabolically active cells using Promega CellTiter-Glo Luminescent Cell Viability Assay (Madison, WI, USA). After the 24-h exposure period, the plate was brought to room temperature and an equal volume of the CellTiter-Glo reagent was added to each well. The plate was then protected from light using foil and placed on an orbital shaker at 10 rpm for 15 min. Full-spectrum luminescence was immediately read using Synergy HTX plate Bio Tek plate reader (Winooski, VT, USA).

##### Mitochondrial Membrane Potential (MMP) Assay

Disruption of mitochondrial membrane potential following exposure to chemical treatments was measured using JC-10 Mitochondrial Membrane Potential Microplate Assay (Abcam, Cambridge, UK). After the 24-h exposure, 50 µL of JC-10 dye loading solution was added to each well and incubated at room temperature for 30 min. An equal of volume of the assay buffer was then added to each well and fluorescent signals were read at 490/525 nm and 540/590 nm using a Synergy HTX plate Bio Tek plate reader (Winooski, VT, USA). The 525 nm emission was then divided by the 590 nm emission to calculated MMP disruption.

##### ROS-Glo Assay

The presence of intracellular H_2_O_2,_ a type of reactive oxygen species, was measured using Promega ROS-Glo Assay (Madison, WI, USA). At 18 h post-treatment, 20 µL of H_2_O_2_ Substrate solution was added to each well and incubated at 37 °C and 5% CO_2_ for an additional 6 h. 100 µL of the ROS-Glo Detection Solution was then added to each well and incubated at room temperature for 20 min, and full spectrum luminescence was read using Synergy HTX plate Bio Tek plate reader (Winooski, VT, USA).

##### NHBE Quality Control and Statistics

Treatment effects for each in vitro assay were investigated using values normalized to vehicle control and were expressed as “% control”. Cell media with 2% DMSO served as the vehicle control and was included on each plate. Menadione (200 µM), which has been shown to induce significant cytotoxicity in NHBE, was used as a positive control for LDH, CTG, and MMP. Coal Tar Extract (SRM 1597a), which has been shown to induce the formation of ROS in NHBE was used as the positive control for ROS-Glo. For CTG and MMP, plates were run in duplicate (n = 6/concentration). For LDH and ROS-Glo single plates were run (n = 4/concentration). Each assay was run at least twice to confirm results. A significance level was defined with a p-value cutoff of 0.05. Significant treatment effects were evaluated relative to vehicle control using one-way ANOVA with Dunnett’s post hoc test.

#### 2.2.2. Zebrafish Maintenance and Exposures

Pathogen-free Tropical 5D wild-type adult zebrafish were housed at approximately 1000 fish per 100 gallons at Sinnhuber Aquatic Research Laboratory (SARL) in accordance with the Institutional Animal Care and Use Committee protocols at Oregon State University (ACUP 2021-0227). Embryos were collected and prepared for exposures as previously described [75,76]. At five to six hours post fertilization (hpf), embryos were robotically loaded into each well of a 96 well plate containing 100 µL of embryo medium. Embryos were statically exposed until 120 h post fertilization. Range finding experiments were conducted with a maximum tested concentration of 600 µM at 1% DMSO for each mixture except Toxicity Mix, which had a maximum concentration of 100 µM (n = 12/concentration). For definitive testing, the maximum tested concentration for Toxicity Mix was 75 µM and 600 µM for other mixtures with triplicate plates (n = 36/concentration).

##### Morphological Assay

Zebrafish were screened for thirteen morphological endpoints at 24 and 120 hpf. At 24 hpf, embryos were assessed for mortality and spontaneous movement (Table S4) [77]. At 120 h post fertilization, embryos were assessed for eleven endpoints which cover range of developmental effects and mortality (Table S4). Images of endpoints measured can be found at the following: https://github.com/Tanguay-Lab/Bioinformatic_and_Toxicological_Resources/tree/main/Files/Zebrafish_Phenotype_Atlas (accessed on 10 October 2022). Evaluation of morphological endpoints was conducted by a formally trained and experienced staff member under a dissecting microscope. Zebrafish data were acquired by the zebrafish acquisition analysis program (ZAAP) which collects developmental endpoints as binary data. To assess the effect of each mixture on zebrafish morphology, percent incidence of morphological abnormalities was calculated across triplicate plates (n = 36/concentration). The percent incidence of any morphological effects or mortality observed in zebrafish was calculated and reported as “% incidence any effect” or “% incidence mortality”. Raw counts for each morphological endpoint can be found in Table S5.

##### Zebrafish Quality Control and Statistics

Negative control wells, DMSO/embryo medium (n = 12) were included on each plate. A positive control (ethyl parathion) plate consisting of a 7-point concentration curve from 4.5–31 uM with n = 12 embryos per concentration was run for daily quality assurance of animal responses. The negative controls had to have ≤20% mortality and morbidity, and the positive control plate had to exhibit an EC_50_ of 19.3 ± 4 µM. Significant morphological effects were assessed in R (version 4.1.2) [78]. Each binary endpoint was recorded for each well as a series of Bernoulli trials (n = 32) as previously described [79]. Binary information was used to evaluate for confounding well, plate, and chemical effects across controls and determine if outliers exist. Outliers were defined as having an incidence greater than three standard deviations from the mean. Significance thresholds for each mixture-endpoint pair were computed based on background incidence for negative control. Fisher’s exact test was used to make comparisons to control, and Bonferroni correction was used to account for family-wise error rate (*p* < 0.01).

#### 2.2.3. Curve fitting and Point of Departure Calculations

Best fit model selection for concentration response curve fit was conducted using the *drc* package in R studio (version 4.1.1) [73]. Best fit models were selected by comparing Akaike information criterion (AIC) scores of six different continuous models included in the *drc* package. For endpoints which no curve could be fit, points of departure were expressed as the lowest effect level (LEL). NHBE MMP and CTG and zebrafish any effect and mortality data showed log-logistic four parameter model, as the model of best fit. Concentration response curves were generated and the concentration at which 50% effect (EC_50_) or mortality (LC_50_) was observed were calculated using GraphPad/Prism (version 6) [80].

## 3. Results

### 3.1. Objective 1: Chemical Prioritization and Mixture Formation

#### 3.1.1. Availability of Toxicity Metrics and Mixture Composition

**Figure 1:**
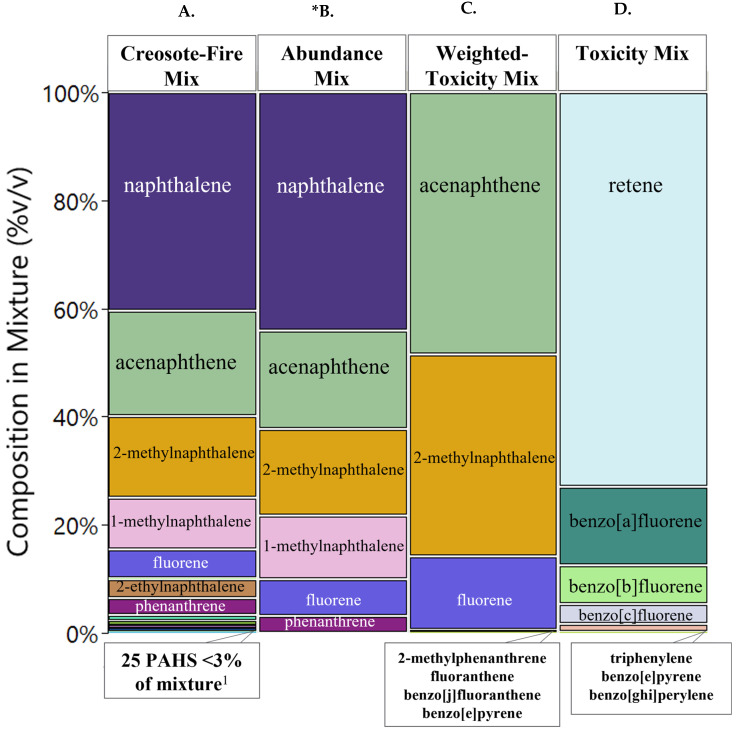
Chemical Composition of Mixtures. Chemicals greater than 3% *v*/*v* in the mixture are shown in colored sections. Colors and area of color correspond to a given chemical and the %v/v of that chemical in its respective mixture. Anything less than 3% *v*/*v* is listed below its respective mixture figure. (**A**). 30 chemicals identified above detection limits out of 63 PAHs air samples were analyzed for. (**B**). Chemicals prioritized strictly based on abundance, top seven PAHs that made up over 97% *v*/*v* in Creosote-Fire Mixture were selected. (**C**). Chemicals prioritized using empirically derived and predicted toxicity metrics. (**D**). Chemicals prioritized by weighting empirical and predicted toxicity metrics with chemical concentrations. ^1^ Full list of chemical composition in each mixture can be found in Tables S9 and S10. * 2-ethylnaphthalene was not included in Abundance Mix due to unavailability of the standard during synthesis of this mixture.

A complex chemical mixture with sources from creosote and wildfire smoke was simplified for hazard identification by prioritizing the most abundant chemical class in the mixture, which were PAHs. 30 PAHs were detected and included in a synthetic mixture called Creosote-Fire Mix. PAHs that made up over 97% of the mixture are shown in Figure 1A. An exposure-based approach, toxicity-based, and combination approach (exposure and toxicity) were explored to further simplify this complex mixture of PAHs. The exposure-based approach prioritized chemical components by selecting the most abundant chemicals in the Creosote-Fire Mix. The top 7 chemicals which made up over 97% of the Creosote-Fire Mix were chosen to create the Abundance Mix (Figure 1B). Naphthalene, acenaphthene, 2-methylnaphthalene, 1-methylnaphthalene, fluorene, phenanthrene, and 2-ethylnaphthalene were prioritized. For consistency, the top 7 ranking chemicals were also selected for the toxicity-based and combination approach.

The toxicity-based (Toxicity Mix) and combination (Weighted-Toxicity Mix) approach both incorporated available toxicity information. Of the 30 PAHs, RfD or RfC was available for a total of 10 (Table 1; Table S6). For cancer-related toxicity values, a total of six OSF or IUR values were available, and 18 IARC classifications were available. RPF values were available for seven of the 30 PAHs [81]. For Weighted-Toxicity Mix TEF values were available for all 30 PAHs. Zebrafish benchmark concentrations were available for 14 of the 30 PAHs.

Toxicity Mix, which largely considered toxicity values in its prioritization procedure, without weighting by chemical abundance (Equation (1)), solely consisted of chemicals in the lower 3% of the Creosote-Fire Mix. This mixture was comprised of less abundant chemicals with greater empirical and QSAR predicted toxicity (Figure 1C). Retene, was the most abundant components in the mixture, followed by benzo(a)fluorene, benzo(b)fluorene, benzo(c)fluorene, triphenylene, and benzo(ghi)perylene. The combination approach considered both hazard and exposure for each chemical in its prioritization approach, by scaling toxicity metrics by chemical concentrations (Equations (2)–(4); Table S7). This mixture, Weighted-Toxicity Mix, prioritized highly abundant chemicals in the Creosote-Fire Mix including acenaphthene, 2-methylnaphthalene, and fluorene (Figure 1D). Less abundant, but more potent, chemicals were also prioritized including 2-methylphenanthrene, fluoranthene, benzo(j)fluoranthene, and benzo(e)pyrene.

#### 3.1.2. Comparison of Toxicity Metrics

**Figure 2:**
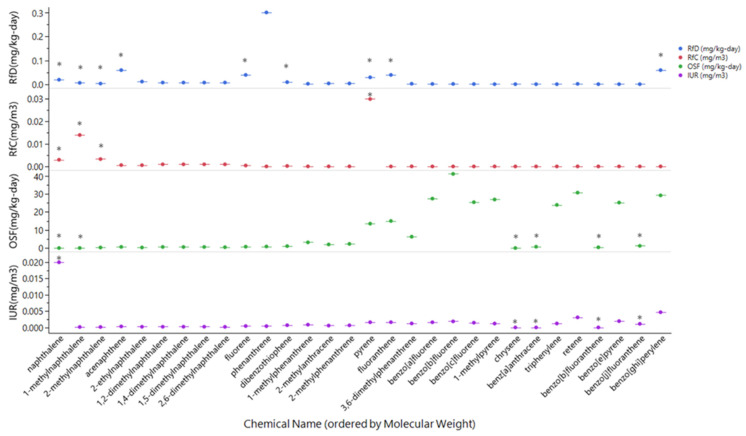
Comparison of Empirically Derived vs. QSAR Predicted Toxicity Values. Predicted RfD and RfC values were within the same magnitude as empirically derived values. IUR predictions were also within the same magnitude as empirical values. OSF had the largest difference between empirical and predicted values, which seemed to be associated with increasing molecular weight. *—empirically derived values. Reference Dose (RfD); Reference Concentration (RfC); Oral Slope Factor (OSF); Inhalation Unit Risk (IUR).

Comparison between empirically derived and QSAR predicted values showed certain toxicity values were better predicted than others (Figure 2). Generally, empirically derived values for RfD and RfC were within the same magnitude of predicted values. IUR predicted and empirically derived values were within the same magnitude of each other. OSF appeared to have the largest difference between empirically derived and predicted values with higher molecular weight PAHs tending to have higher, more toxic estimates.

**Figure 3:**
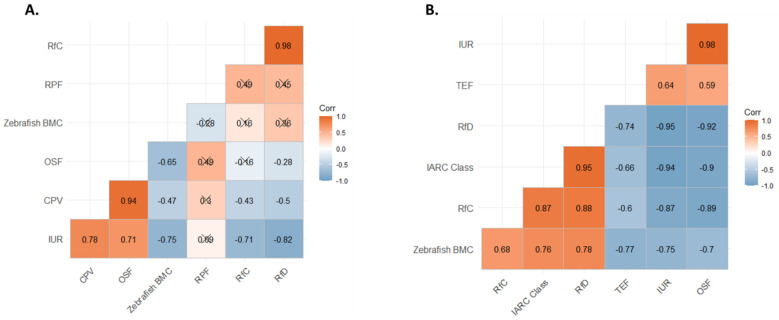
Correlations of Toxicity Metrics. (**A**) Correlation matrix of rankings for toxicity metrics used for Toxicity Mix. (**B**) Correlation matrix of toxicity metric rankings for Weighted-Toxicity Mix. Each square is labeled with correlation coefficient. Anything with an X was not significant. Significance cut-off was *p* < 0.05 using Pearson correlation coefficient. Correlations were conducted for each chemical only for complete observations.

Correlations of chemical rankings were investigated to identify similarities or differences between chemical prioritization of the different toxicity metrics (Table S8). For the toxicity-based approach (Toxicity Mix) significant positive correlations were observed between most toxicity metrics, except for RPF. RfC and RfD had the strongest correlation of 0.98 followed by CPV and OSF with a value of 0.94 (Figure 3A). A moderate correlation was observed between zebrafish BMC and RfD with a p-value of 0.07. Correlations were separated into non-cancer or cancer-based metrics. For the combination approach (Weighted-Toxicity Mix), significant correlations were observed between all toxicity metric rankings (Figure 3B). Like Toxicity Mix metrics, positive correlations were observed between two distinct groups. Cancer-relevant toxicity values IUR/OSF and TEF were positively correlated with each other. Non-cancer toxicity metrics were also positively correlated with each other. This included IARC classification, Zebrafish BMC, RfD and RfC. Significant negative correlations were observed overall between non-cancer and cancer-based metrics.

### 3.2. Objective 2: Comparison of Mixture Toxicity

#### 3.2.1. NHBE Bioactivity Screening and Mixture Potency

**Figure 4:**
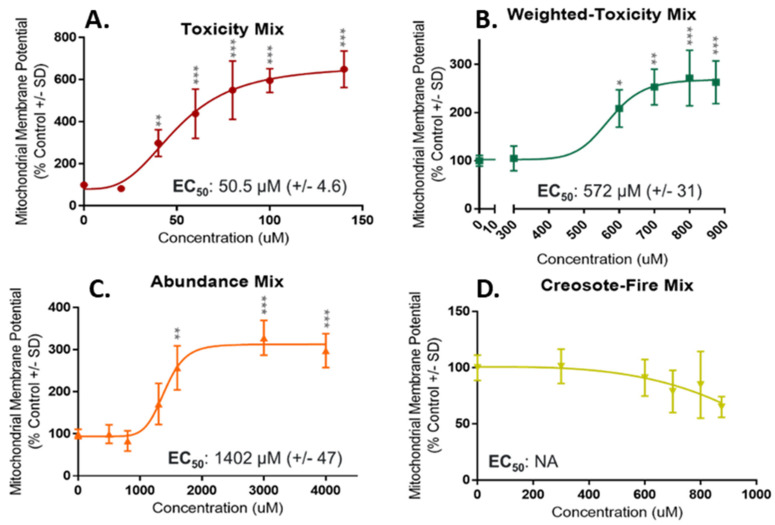
Concentration-Response Curves for Mitochondrial Membrane Potential in NHBE. Curves are in order of decreasing potency based on predicted EC_50_ values. (**A**) Toxicity Mix was the most potent mixture with an EC_50_ of 50.5 µM. (**B**) Weighted-Toxicity Mix was the second most potent mixture with an EC_50_ of 572 µM. (**C**) Abundance Mix was the least potent mixture with EC_50_ of 1402 µM. (**D**) Creosote-Fire Mix did not elicit significant bioactivity compared to control, no EC_50_ was calculated. Concentrations significantly different from control are denoted with an asterisk (*). *p* < 0.05 *; *p* < 0.01 **; *p* < 0.001 ***.

**Figure 5:**
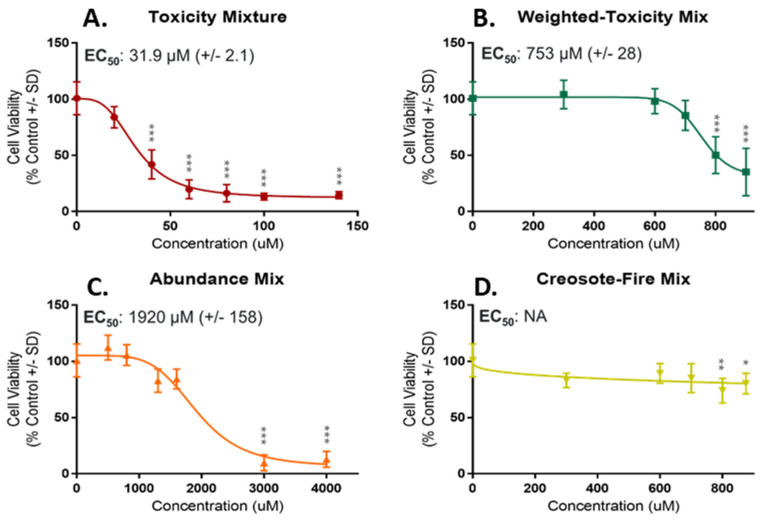
Concentration-Response Curves for Cell Viability in NHBE. Curves are in order from high to low potency based on predicted EC_50_ values. (**A**) Toxicity Mix was the most potent mixture with an EC_50_ of 31.9 µM. (**B**) Weighted-Toxicity Mix was the second most potent mixture with an EC_50_ of 753 µM. (**C**) Abundance Mix was the least potent mixture with EC_50_ of 1920 µM. (**D**) Creosote-Fire Mix had significant bioactivity for two highest doses, however, no EC_50_ was calculated. Concentrations significantly different from control are denoted with an asterisk (*). *p* < 0.05 *; *p* < 0.01 **; *p* < 0.001 ***.

Toxicity Mix, Weighted-Toxicity Mix and Abundance Mix were bioactive for mitochondrial membrane potential and cell viability (Figure 4 and Figure 5). Bioactivity was also observed for Toxicity Mix and Abundance Mix for ROS generation (Figure S1). For release of LDH, significant bioactivity was observed for Weighted-Toxicity Mix and Abundance Mix (Figure S2; Table S11). Toxicity Mix was the most potent mixture with tested concentrations ranging from 20 to 140 µM for MMP and cell viability. Significant concentrations for MMP and cell viability ranged from 40 to 140 µM and respective EC_50_ values of 50.5 µM (±4.6) and 31.9 µM (±2.1). For ROS generation a curve was not able to be fit, however Toxicity Mix had a LEL of 20 µM. Weighted-Toxicity Mix was the second most potent mixture, with concentrations ranging from 300 to 900 µM for MMP and cell viability. Significant concentrations for MMP ranged from 600 to 900 µM with an EC_50_ value of 572 µM (±31). For cell viability, significant concentrations ranged from 800 to 900 µM with an EC_50_ value of 753 µM (±28). For release of LDH an EC_50_ value of 798 µM (±60.5) was observed. Of the bioactive mixtures, Abundance Mix was the least potent mixture with bioactive concentrations ranging from 500 to 4000 µM. For MMP, significant concentrations ranged from 1600 to 4000 µM with an EC_50_ value of 1402 µM (±47). Significant concentrations for cell viability ranged from 3000 to 4000 µM with an EC_50_ value of 1920 µM (±158). For release of LDH, Abundance Mix had an EC_50_ value of 2095 µM (±380). For ROS generation a LEL of 500 µM was observed.

#### 3.2.2. Zebrafish Bioactivity Screening and Mixture Potency

**Figure 6:**
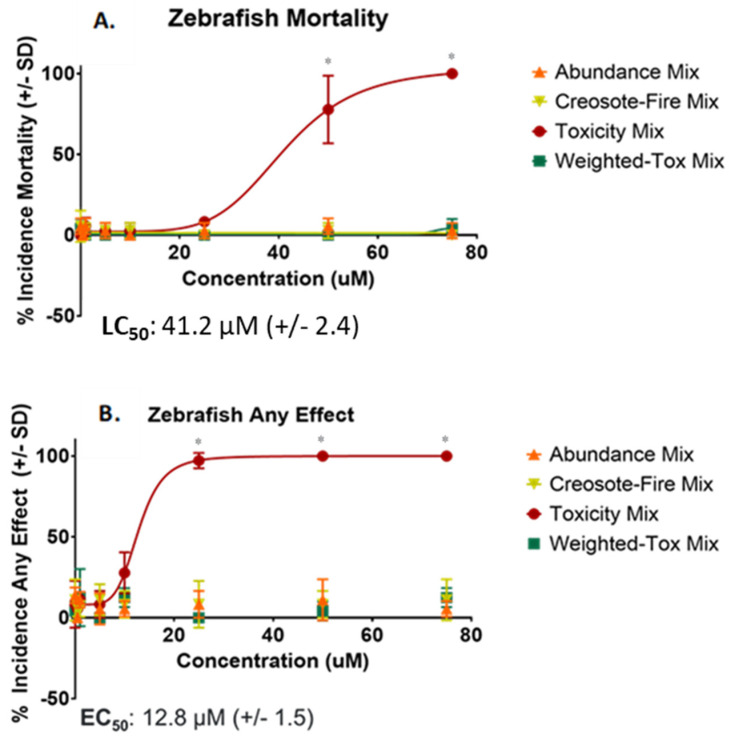
Concentration-Response Curves for Mortality and Any Effect in Zebrafish. (**A**) Percent incidence of Zebrafish mortality and predicted LC_50_ and (**B**) Percent incidence of any effect and predicted EC_50_ value. Toxicity Mix was the only mixture with significant bioactivity in zebrafish. Any concentration at or above binomial significance threshold are denoted with an asterisk (*).

In zebrafish, mortality and the suite of morphological endpoints summarized as ‘any effect’ were investigated. Toxicity Mix was the most potent, and only mixture with statistically significant bioactivity, at test concentrations ranging from 1 to 75 µM (Table S12). Toxicity Mix had significant effects for mortality starting at 50 µM with an EC_50_ value of 41.2 µM (±2.4) (Figure 6A). For ‘any effect’, significant effects were observed starting at 25 µM with an EC_50_ value of 12.8 µM (±1.5) and were mainly associated with craniofacial defects or edemas (Figure 6B). Due to the observed differences in potency between Toxicity Mix and the other mixtures in NHBE, a second round of screening was conducted for Weighted-Toxicity Mix, Abundance Mix and Creosote-Fire Mix at higher concentrations (200 to 600 µM). However, no significant bioactivity or concentration-response effects were observed (Figure S3).

## 4. Discussion

### 4.1. Chemical Prioritization Appraoches

There are many available approaches to create sufficiently similar mixtures. How these mixtures are formed can significantly impact chemical composition and toxicity results [32]. Prioritization of individual chemicals for mixture toxicity testing have been focused on the exposure or biological effects of chemicals. Exposure-based prioritization is most used to form sufficiently similar mixtures. After prioritization of chemicals, proportions in the mixtures are based on median or average environmental exposure concentrations from multiple samples [10,33,82]. Biological effect-based studies have prioritized chemicals most commonly for representative mixture formations [1,6,13,14,15]. These studies are often not based on real-world environmental mixtures and are designed to investigate specific interactions between chemicals when combined in a mixture. Chemical proportions within the mixture are formed based on effective concentration levels from screening individual components or existing toxicity information [34,83]. Some studies have incorporated environmental and toxicological information for chemical prioritization for mixture toxicity testing [13,17,84]. For mixtures formed in these studies chemicals were retained at their environmental proportions.

In this study, three different approaches based on the methodology described above were used to form sufficiently similar mixtures from the same complex mixture of interest. Comparisons between chemical composition and observed biological effects based on mixture formation were made. Complex mixtures of organic and inorganic chemicals related to contamination from historic use of creosote and wildfire smoke impact have been reported [85,86,87,88,89]. In order to simplify this complex environmental mixture, samples were analyzed for PAHs due to their high prevalence from exposure sources at the sampling site [44,45,90]. Traditionally, environmental studies have used the 16 priority PAHs established by U.S. EPA in the 1970s as the standard for investigations [91,92]. This consists of unsubstituted PAHs that were prioritized based on availability of analytical standards, occurrence in the environment, and known toxicity [93]. However, PAHs are a class of over 100 different chemicals [94]. In this study, samples were analyzed for a total of 63 unsubstituted and alkylated PAHs, with approximately 20 being alkylated PAHs. The presence of unsubstituted PAHs and the associated toxicity have been widely studied [12]. However, there has been less emphasis on alkylated PAHs despite evidence that alkyl PAHs may possess toxicity that surpasses their parent compounds [92]. Of the 30 PAHs identified in the environmental samples, 13 of them were alkylated PAHs. Three of the PAHs that accounted for 97% of the total concentration of the Creosote-Fire Mix were alkyl PAHs. However, additional published and unpublished studies at this site using an alkylated PAH-specific method identified a high abundance of alkyl PAHs [58]. This suggests that alkyl PAHs may be underrepresented in our 30 PAH mixture due to the chemical analysis method used for this sample.

### 4.2. Comparison and Availability of Toxicity Values

Due to the large availability of toxicity information for individual chemicals from in vitro, in vivo, or in silico models, recent studies have begun integrating multiple lines of evidence to prioritize chemicals of interest [12,13,17,35]. During collection of toxicity values for this study, a major data gap regarding available toxicity information for PAHs was identified. The use of this approach for Toxicity Mix and Weighted-Toxicity Mix allowed for a more holistic view of the chemicals without being limited based on available toxicity information or specific biological endpoints. RfD had the largest number of available empirically derived toxicity values, which only accounted for 30% of the PAHs in the Creosote-Fire Mix. To fill these data gaps, a QSAR model which predicted toxicity values based on empirically derived toxicity values was incorporated. Overall, this model could predict toxicity values within the same magnitude of empirically derived values for the non-cancer toxicity values (RfD/RfC). There was less certainty in the estimate for cancer-based toxicity values, with increasing distance in the magnitude of empirically derived vs. predicted values with increasing molecular weight. A relationship between molecular weight and carcinogenicity has been established, which may explain the QSAR model predicting higher OSF values for higher molecular weight PAHs [95,96]. Further investigation into the chemicals used to build this model showed that of the 886 chemicals used in the model approximately 19 were PAHs. Of the 19 PAHs, only four were alkylated PAHs [67]. This tool provided incredibly useful information to address missing toxicity values that were essential for the workflow of this study. However, these findings highlight an important area of additional research that is needed for PAHs, or more broadly polycyclic aromatic compounds (PACs). Additional toxicity information on a more structurally diverse set of PACs, may not only help supplement the prioritization of hazardous PACs at a contaminated site, but can also improve predictive models such as the one used in this study. Overall, this study observed a general lack of toxicity information particularly for substituted PAHs, such as alkylated PAHs [12]. This has also been identified as a research priority by the National Toxicology Program.

Correlations between how toxicity metrics prioritized individual PAHs were explored. The Toxicity Mix and Weighted-Toxicity Mix were formed using an iterative process. Some toxicity metrics used for Toxicity Mix were changed for the prioritization of components for Weighted-Toxicity Mix. Information provided by CPV and OSF were seen to be redundant, which can be seen by the high correlation coefficient (Figure 3A). Further investigation of this did show the same value was reported for some compounds. CPV is the California EPA’s version of OSF which explains why the same values were observed for some chemicals [67]. Additionally, RPF was replaced with TEF due to the lack of information for the PAHs in our mixture from RPF. TEF values were able to provide toxicity information for all the PAHs in the complex mixture. However, we observed that many of the PAHs in our mixture had the same value for TEF resulting in a larger influence of chemical concentration when toxicity metrics were weighted (Table S6). IARC Classification was also included to expand toxicity information used for chemical prioritization. IARC Classifications were available for 18 of the 30 PAHs in the Creosote-Fire Mix, five of these were classified as Group 2B carcinogens by IARC, or possibly carcinogenic to humans.

For both Toxicity Mix and Weighted-Toxicity Mix approaches, traditional non-cancer toxicity values, RfC and RfD, and cancer-based toxicity values, OSF and IUR, were significantly correlated with each other. Since these toxicity values are derived similarly, but for different routes of exposure, strong correlations between the two metrics were expected [97]. IARC class also had strong correlations with RfD. Most of the chemicals detected in our environmental sample were class 2B or 3, possibly carcinogenic to humans or not classifiable as carcinogens to humans [69]. Because these chemicals had low carcinogenic potential, this may explain why they were more closely related to rankings from non-cancer metrics rather than cancer-based values. Significant or moderately significant correlations were also observed between zebrafish BMC ranking and traditional toxicity values, RfC/RfD. These results provide insight into the utility of using high-throughput, non-mammalian invertebrate models for general hazard assessment of chemicals.

### 4.3. Comparison of Mixture Composition and Toxicity

The goal of the exposure-based approach was to prioritize the most abundant chemicals in the Creosote-Fire Mix. The most abundant chemicals were chosen by selecting chemicals that made up over 97% of the total concentration of the mixture. This resulted in seven PAHs being selected. The composition of Abundance Mix consisted mostly of priority PAHs except for the alkylated naphthalenes: 1-methyl- and 2-methylnaphthalene. Bioactivity screening of this mixture in NHBE identified impacts of Abundance Mix on mitochondrial membrane potential, cell viability, release of LDH, and ROS generation at high concentrations, with EC_50_ values of 1402, 1920, 2097 µM, and an LEL of 500 µM, respectively. This highlights the relatively low potency of this mixture regarding cytotoxicity. Additionally, no bioactivity was observed in vivo in early life-stage zebrafish. Similar mixture formation approaches have shown little bioactivity particularly at environmentally relevant concentrations [98,99]. However, the composition of the mixture and exposure concentrations are stronger predictors of biological activity than approach. Our study implemented a range of doses in order to capture a significant biological effect which has been recommended for mixture studies [14]. Considering the toxicity of individual chemicals within this mixture, these results are fairly consistent with previous studies with no notable impacts on cytotoxic endpoints in a number of in vitro studies for naphthalene, 1- or 2-methylnaphthalene [65,91,100]. Studies investigating cytotoxicity of acenaphthene, phenanthrene and fluorene have conflicting results regarding impacts for cytotoxic endpoints [100]. While most in vitro studies noted no significant impacts for naphthalene and 1- and 2-methynaphthalene, rodent assays have identified these chemicals as having impacts on the lung [101,102,103]. Naphthalene is also classified as a possible carcinogen in humans [69].

The toxicity-based approach was intended to select the most hazardous components in the Creosote-Fire Mix. Toxicity Mix was formed using chemical abundance, empirically derived and QSAR predicted toxicity values, zebrafish screening data, and relative potency factors. Incorporation of this toxicity information enabled the prioritization of seven components of most concern in the Creosote-Fire Mix based on available data. This was confirmed during bioactivity screening with Toxicity Mix being the most potent mixture with respective EC_50_ values in NHBE of 31.9 and 50.5 µM, and an LEL of 20 µM for mitochondrial membrane potential, cell viability, and ROS generation. Toxicity Mix was also the only mixture to elicit significant biological effects in zebrafish. The toxicity results for this mixture are consistent based on available studies for individual chemicals in Toxicity Mix. Retene, benzo[c]fluorene, benzo(e)pyrene, and benzo[ghi]perylene have all been reported to have impacts on cytotoxic endpoints and have mutagenic properties [91,100,104,105,106]. Specifically, benzo[c]fluorene has been reported to induce tumor formation in mice. Traditional toxicity-based mixtures have typically focused on specific biological endpoints when prioritizing chemical components for the mixture of interest [34,83]. In this study, we incorporated a more holistic view of toxicity including information for cancer and non-cancer endpoints and chemical abundance as individual measures for prioritization. This approach utilizing database and QSAR-predicted toxicity values enabled rapid chemical prioritization in our mixture of interest and provided an alternative way to prioritize chemicals without generating new toxicity data.

The combination approach used to form Weighted-Toxicity Mix was intended to prioritize high abundance and high hazard chemicals in the Creosote-Fire Mix. Scaling toxicity values by environmental concentrations for chemical prioritization for mixture toxicity studies has not been widely implemented. However, the importance of prioritizing chemicals by both exposure and biological hazard is recognized by the exposure science and toxicology community [6,13,17]. Weighted-Toxicity Mix was formed using weighted empirically derived and QSAR predicted toxicity values, zebrafish screening data, IARC cancer classification, and toxic equivalency factors. Using this approach, chemicals high in abundance and hazard were prioritized. Acenaphthene, fluorene and 2-methylnaphthalene were the most abundant chemicals in Weighted Toxicity Mix, which were also prioritized using the exposure-based approach. Some differences were observed between components prioritized by toxicity-based and combination approaches likely due to the scaling of the toxicity metrics and incorporation of additional toxicity information for this approach. One chemical, benzo[e]pyrene was included in both mixtures. Weighted-Toxicity Mix was the second most potent mixture, however EC_50_ values were much higher than those observed in the Toxicity Mix. Respective EC_50_ values for Weighted-Toxicity Mix were 572, 753, and 773 µM for mitochondrial membrane potential, cell viability, and release of LDH. These results are consistent with observed effects noted previously with little bioactivity related to acenaphthene, fluorene, 2-methylnaphthalene, and benzo[j]fluoranthene. However, higher potency may be due to the addition of the lower concentration chemicals 2-methylphenanthrene, benzo(e)pyrene, and fluoranthene which have been reported as being bioactive for cytotoxic endpoints in in vitro studies [91,100,104,107].

### 4.4. Mixture Formation Implications and Potential Mixture Interactions

Overall, similarities and differences were observed in chemical composition, bioactivity, and potency based on mixture formation approach. When only considering abundance for chemical prioritization (Abundance Mix), chemicals which were found at the highest observed concentrations in our mixture of interest were prioritized. This mixture was the least potent of the other mixtures, and only elicited an effect at high test concentrations. When prioritizing components almost solely based on toxicity information (Toxicity Mix), chemicals at relatively low environmental concentrations were prioritized. Although these chemicals are present at lower environmental concentrations, lower concentrations of these chemicals induced an effect. Additionally, this mixture consisted entirely of non-priority PAHs, further highlighting the need for alternative methods to assess toxicity of understudied chemicals in environmental investigations. Identification of these non-priority PAHs in the Toxicity Mix, as potential drivers of toxicity, supports the need for further investigation into this mixture, as potential interactions could contribute to higher potency and therefore greater toxicity.

The last approach, which incorporated hazard and abundance (Weighted-Toxicity Mix), by scaling toxicity information with environmental concentrations, resulted in prioritization of chemicals at higher concentrations and chemicals at lower concentrations, but of higher hazard. There was overlap with the highly abundant chemicals in this mixture and Abundance Mix. However, biological activity and potency cannot be predicted by composition alone [24]. Due to the overlap between chemical components and proportions in Abundance Mix and Weighted-Toxicity Mix similarities in potency between the two mixtures may have been expected. However, the EC_50_ of cytotoxic endpoints for Weighted-Toxicity Mix were approximately half of those observed with the Abundance Mix. Therefore, it is expected that the less abundant, more potent chemicals, are driving the observed toxicity in this mixture. Specifically, interactions between less abundant chemicals may be occurring. Studies have shown that when added to complex mixtures or combined with known carcinogens fluoranthene and benzo[e]pyrene have been shown to increase toxicity [108,109]. Alternatively, a decrease in toxicity has also been observed when fluoranthene and benzo[e]pyrene were combined with other combinations of PAHs or complex mixtures [108,110]. In the case of Weighted-Toxicity Mix, it is believed that the addition of fluoranthene and benzo[e]pyrene may increase the mixture’s toxicity. Interestingly, while all sufficiently similar mixtures demonstrated some bioactivity in vitro, the Creosote-Fire Mixture did not have any notable toxicity. The low potency of this mixture for the endpoints in this study may be attributed to constraints regarding test concentration due to the complexity of the mixture. Due to the high number of chemical components in this mixture we were constrained by limits of solubility and were not able to test at concentrations as high as the Abundance Mix, which accounted for a significant portion of the total concentration of Creosote-Fire Mix. Additionally, low potency could also be due to unknown mixture interactions that could be occurring between individual chemicals in this mixture. As previously noted, differences in toxicity have been observed when PAHs have been combined into complex mixtures. Studies have shown when mutagenic compounds were exposed with complex mixtures a decrease in potency was observed which may be a result of competitive inhibition preventing the formation of reactive metabolites [110,111,112]. Similar results have also shown decreasing mutagenic activity with increasing mixture complexity of PAHs [113]. Although increase in potency has also been observed [111,112]. While there are no known carcinogens in this study, it is possible that some of the more potent chemicals, such as those in the Toxicity Mix that have been shown to have mutagenic properties, may have reduced potency when combined with other PAHs in the Creosote-Fire Mix due to interactions with these other chemicals. Mixture interactions that may be occurring within these mixtures highlight the complexity of mixtures and the different interactions that may occur depending on the composition of the mixture [113].

## 5. Conclusions

Currently, there is no guidance as to which components need to be in common between the sufficiently similar and mixture of interest. This lack of guidance results in several different approaches to form sufficiently similar mixture that may cause differences in toxicity screening results. This study explored different approaches to form sufficiently similar mixtures to make comparisons between chemical composition, bioactivity, and potency of mixtures based on the approach. The toxicity-based and novel combination approach used multiple lines of evidence to fill data gaps for existing toxicity data and account for a wide array of biological activity of PAHs. Through this process significant data gaps regarding available toxicity information for PAHs was identified. To address these data gaps, in silico models were used, however, gaps in toxicity information impacted the accuracy of in silico predicted toxicity values. Toxicity information for alkylated PAHs was particularly sparse highlighting the need for more toxicity studies on this subset of PAHs. Comparison of high-throughput screening in zebrafish did show similar prioritization of chemicals in this study with traditional non-cancer toxicity values, which highlights the value of zebrafish for prioritization of PAHs that may be further investigated for the derivation of toxicity values.

The results from this study provide useful information regarding potential approaches used for simplifying complex mixtures for hazard characterization. Results demonstrated the combination approach as the ideal approach due its ability to prioritize chemicals with high exposure and hazard potential. In this study, direct toxicological comparisons were not able to be made between the complex mixture and the simple mixtures to evaluate for sufficient similarity from a biological standpoint. Future studies could further investigate the utility of this weighted approach against a more well-characterized complex mixture (i.e., a standard reference mixture) rather than the real-world mixture with unknown toxicity used in this study. Results showed Toxicity Mix was the most potent mixture, although the chemicals in this mixture are understudied. These results warrant further investigations into the toxicity of the chemicals in Toxicity Mix. Additionally, differences in potency between the Abundance and Weighted-Toxicity Mix, regardless of the significant overlap in chemical composition, were observed. These results also suggest future studies could investigate the individual chemicals in Weighted-Toxicity Mix to identify which chemicals may be contributing to the higher potency of this mixture.

## Figures and Tables

**Table 1:** Collected toxicity metrics, abbreviations and associated sources.

Toxicity Metrics	Abbreviation	Sources *
Reference Concentration	RfC	IRIS [64]; EPA Comptox Chemicals Dashboard [65]; CTV [67]
Reference Dose	RfD	IRIS [64]; EPA Comptox Chemicals Dashboard [65]; CTV [67]
Inhalation Unit Risk	IUR	California EPA [72]; EPA Comptox Chemicals Dashboard [65]; CTV [67]
Oral Slope Factor	OSF	California EPA [72]; EPA Comptox Chemicals Dashboard [65]; CTV [67]
Cancer Potency Value	CPV	IRIS [64]; CTV [67]
Relative Potency Factor	RPF	IRIS [64]
Toxic Equivalency Factor	TEF	Samburova 2017 [70]
IARC Classification	IARC Class	IARC [69]
Zebrafish Benchmark Concentration	Zfish BMC	Shankar et al., 2019 [68]

* Toxicity values were prioritized using the following criteria IRIS > EPA PPRTV > EPA > CalEPA > ATSDR > HEAST> Regional EPA > non-EPA.

## Data Availability

Not applicable.

## Supplementary Materials

The following supporting information can be downloaded at: https://www.mdpi.com/article/10.3390/toxics10110651/s1, Table S1: List of polycyclic aromatic hydrocarbon CAS#’s, detection limits, and physicochemical properties in analytical method used to quantify environmental samples and sufficiently similar mixtures in order of retention time. Table S2: PAH concentrations (ng/m3) for each site and mean and standard deviation. Table S3: List of chemicals used to create sufficiently similar mixtures, purity, and supplier. Table S4: Key to teratogenic super endpoint abbreviations with definitions. Table S5: ncidence (%) of effect for 13 zebrafish morphological endpoints for Abundance Mix (Abun. Mix), Creosote-Fire Mix (SC Mix), Toxicity Mix (Tox Mix) and Weighted Toxicity Mix (WA-Tox Mix) and for A.) 0–75 μM and B.) 0–600 μM test concentrations (conc). Abbreviations for each morphological endpoint can be found in Table S4. Table S6: Key to teratogenic super endpoint abbreviations with definitions. Table S5: ncidence (%) of effect for 13 zebrafish morphological endpoints for Abundance Mix (Abun. Mix), Creosote-Fire Mix (SC Mix), Toxicity Mix (Tox Mix) and Weighted Toxicity Mix (WA-Tox Mix) and for A.) 0–75 μM and B.) 0–600 μM test concentrations (conc). Abbreviations for each morphological endpoint can be found in Table S4. Table S6: List of collected toxicity metrics for the detected PAHs in the environmental sample and associated sources for each toxicity metric. Empirically derived values are in bold for Cancer Potency Value (CPV), Inhalation Unit Risk (IUR), Reference Concentration (RfC), Oral Slope Factor (OSF), and Reference Dose (RfD). Table S7: Rankings of chemicals for individual toxicity metrics for Toxicity Mixture. Table S8: Ranking of chemicals for individual toxicity metrics for Weighted-Toxicity Mixture. Table S9: Percentage of chemicals in each sufficiently similar mixture. Table S10: Percentage of chemicals in 30 PAH mixture (Creosote-Fire Mixture). Table S11: Bioassay results in 2D NHBE expressed as average percent response relative to vehicle control +/- standard deviation. Average concentrations calculated for total of n=6 from two duplicate plates for MMP and CTG. Average concentrations for total n = 4 from a single plate for ROS-Glo and LDH. Concentration not tested for a given assay were denoted by NA. Table S12: % Incidence of zebrafish endpoints any effect and mortality at 120 hours post fertilization (hpf). Average % incidence from 3 replicate plates with n = 12 on each plate for a total n = 36. Each mixture had its own set of controls for each plate. Figure S1: Concentration-Response Curves for Release of LDH in NHBE. Plots are in order of decreasing potency based on predicted EC50 values. Concentrations significantly different from control are denoted with an asterisk (*). *p* < 0.05 *; *p* < 0.01 **; *p* < 0.001 *** Figure S2: ROS generation in NHBE and associated lowest effect levels (LEL). LEL defined as the lowest concentration significant from control. Concentrations significantly different from control are denoted with an asterisk (*). *p* < 0.05 *; *p* < 0.01 **; *p* < 0.001 *** Figure S3: Concentration response plots for zebrafish morphology screening for the positive control (Parathion), Abundance Mix (Abun. Mix), Creosote-Fire Mix (SC Mix), Toxicity Mix (Tox Mix) and Weighted Toxicity Mix (WA-Tox Mix) and for A.) 0–75 μM and B.) 0-600 μM test concentrations. Morphological effects above the binomial significance threshold are denoted with a red circle. Each red circle represents an individual hit for that morphological effect. Reference [114] is cited in the supplementary materials.
