# Supplementary material for: Leveraging Multiple Data Streams for Prioritization of Mixtures for Hazard Characterization
Source: Toxics. 2022 Oct 29;10(11):651. doi: 10.3390/toxics10110651 (PMC9699527; doi:10.3390/toxics10110651)
Supplement: Supplementary file 1 [file toxics-10-00651-s001.zip › toxics-1935998-supplementary-2022.11.15 ╕n╨┬ (2).pdf]

Supporting Information for Leveraging Multiple Data Streams for Prioritization of Mixtures for Hazard Characterization

Brianna N. Rivera [1], Christine C. Ghetu [1], Yvonne Chang [1], Lisa Truong [1], Robyn L. Tanguay [1], Kim A. Anderson [1], Susan C. Tilton [1]

[1]Department of Environmental and Molecular Toxicology, Oregon State University, Corvallis, OR 97331, USA

Table of Contents:

Figures: **Page Number**

S1: Concentration-Response Curves for Release of LDH in NHBE. 12

S2: ROS generation in NHBE and associated lowest effect levels (LEL). 13

S3: Concentration response plots for zebrafish morphology screening for the positive control… 17

Tables:

S1: List of polycyclic aromatic hydrocarbon CAS#'s, detection limits, and physicochemical… 1

S2: PAH concentrations (ng/m3) for each site and mean and standard deviation. 3

S3: List of chemicals used to create sufficiently similar mixtures, purity, and supplier. 4

S4: Key to teratogenic super endpoint abbreviations with definitions 5

S5: Incidence (%) of effect for 13 zebrafish morphological endpoints for Abundance Mix… 6

S6: List of collected toxicity metrics for the detected PAHs in the environmental sample… 8

S7: Rankings of chemicals for individual toxicity metrics for Toxicity Mixture 9

S8: Ranking of chemicals for individual toxicity metrics for Weighted-Toxicity Mixture 10

S9: Percentage of chemicals in each sufficiently similar mixture. 11

S10: Percentage of chemicals in 30 PAH mixture (Creosote-Fire Mixture) 11

S11: Bioassay results in 2D NHBE expressed as average percent response relative to… 13

S12: % Incidence of zebrafish endpoints any effect and mortality at 120 hours post fertilization.... 14

Table S1. List of polycyclic aromatic hydrocarbon CAS#'s, detection limits, and physicochemical properties in analytical method used to quantify environmental samples and sufficiently similar mixtures in order of retention time.

Chemical Name	CAS #	LOD (pg/uL)	LOQ (pg/uL)	MW (g/mol)	log Koa[1]	log Kow[2]	H298 (atm*m3*mole-1)[3]
fluorene-d10	81103-79-9	.33	1	176.18	6.58	4.18	1.67E-04
benzo[b]fluoranthene-d12	205-99-2	1.67	5	264.15	10.35	5.78	8.10E-07
pyrene-d10 - PRC	1718-52-1	0.42	2.09	212.12	8.19	4.88	8.30E-06
naphthalene	91-20-3	1.04	5.20	128.17	5.05	3.3	5.26E-04
2-methylnaphthalene	91-57-6	0.70	3.50	142.2	5.53	3.86	5.80E-04
1-methylnaphthalene	90-12-0	0.28	1.39	142.2	5.55	3.87	5.80E-04
2-ethylnaphthalene	939-27-5	0.97	4.84	156.09	6.04	4.38	7.71E-04
2,6-dimethylnaphthalene	581-42-0	0.89	4.43	156.22	5.89	4.31	6.41E-04
1,4-dimethylnaphthalene	571-58-4	1.24	6.22	156.22	6.17	4.37	6.41E-04
1,5-dimethylnaphthalene	571-61-9	1.19	5.93	156.22	6.22	4.38	6.41E-04
1,2-dimethylnaphthalene	573-98-8	.94	4.70	156.22	5.89	4.31	6.41E-04
1,8-dimethylnaphthalene	569-41-5	0.83	4.15	156.22	6.22	4.26	6.41E-04
2,6-diethylnaphthalene	59919-41-4	0.81	4.06	184.27	6.59	**5.25**	1.13E-03
acenaphthylene	208-96-8	2.33	11.65	152.19	6.27	3.94	5.48E-05
acenaphthene	83-32-9	1.07	5.35	154.2	6.04	3.92	2.82E-04
fluorene	86-73-7	0.79	3.97	166.22	6.59	4.18	1.67E-04
dibenzothiophene	132-65-0	0.24	1.20	184.26	7.24	4.38	2.79E-05
phenanthrene	85-01-8	0.46	2.31	178.23	7.22	4.46	5.13E-05
anthracene	120-12-7	1.05	5.23	178.23	7.09	4.45	5.13E-05
2-methylphenanthrene	2531-84-2	0.39	1.93	192.25	7.50	4.86	5.67E-05
2-methylanthracene	613-12-7	0.47	2.36	192.25	7.64	5	5.67E-05
1-methylphenanthrene	832-69-9	1.06	5.32	192.25	7.78	5.08	5.67E-05
9-methylanthracene	779-02-2	0.87	4.37	192.25	7.87	5.07	5.67E-05
3,6-dimethylphenanthrene	1576-67-6	0.42	2.08	206.28	8.03	**5.44**	6.25E-05
fluoranthene	206-44-0	0.54	2.72	202.26	8.60	5.16	8.30E-06
2,3-dimethylanthracene	613-06-9	0.34	1.71	206.28	8.03	**5.44**	6.25E-05
9,10-dimethylanthracene	781-43-1	0.85	4.23	206.28	8.28	5.69	6.25E-05
pyrene	129-00-0	0.42	2.09	202.25	8.19	4.88	8.30E-06
retene	483-65-8	0.84	4.19	234.33	8.70	**6.35**	1.10E-04
benzo[a]fluorene	238-84-6	1.67	5	216.23	8.36	5.4	1.63E-05
benzo[b]fluorene	243-17-4	1.67	5	216.23	9.57	5.77	1.63E-05
benzo[c]fluorene	205-12-9	0.30	1.50	216.23	8.367	**5.19**	1.63E-05
1-methylpyrene	2381-21-7	0.38	1.90	216.28	8.91	**5.48**	9.16E-06
benz[a]anthracene	56-55-3	0.75	3.77	228.29	9.0	5.76	5.01E-06
cyclopenta[cd]pyrene	27208-37-3	0.53	2.67	226.27	10.15	**5.7**	8.65E-07
triphenylene	217-59-4	0.41	2.04	228.29	10.69	5.49	5.01E-06
chrysene	218-01-9	0.50	2.49	228.28	9.48	5.81	5.01E-06
6-methylchrysene	1705-85-7	0.89	4.44	242.31	9.72	**6.07**	5.53E-06

5-methylchrysene	3697-24-3	1.67	5	242.31	9.72	**6.07**	5.53E-06
benzo[b]fluoranthene	205-99-2	0.37	1.85	252.3	10.35	5.78	8.10E-07
7,12-dimethylbenz[a]anthracene	57-97-6	0.94	4.71	256.34	9.61	5.8	6.10E-06
benzo[k]fluoranthene	207-08-9	0.53	2.63	252.3	10.73	6.11	8.10E-07
benzo[j]fluoranthene	205-82-3	0.56	2.79	252.3	10.59	**6.11**	8.10E-07
benzo[e]pyrene	192-97-2	0.71	3.53	252.3	11.35	6.44	8.10E-07
benzo[a]pyrene	50-32-8	1.18	5.90	252.3	10.86	6.13	8.10E-07
indeno[1,2,3-cd]pyrene	193-39-5	0.26	1.32	276.33	11.55	**6.7**	1.31E-07
dibenzo[a,h]anthracene	53-70-3	1.02	5.11	278.35	11.78	6.75	4.89E-07
benzo[a]chrysene	213-46-7	0.74	3.72	278.35	11.81	7.11	4.89E-07
benzo[ghi]perylene	191-24-2	0.34	1.71	276.33	11.50	6.63	1.31E-07
anthanthrene	191-26-4	0.33	1.65	276.33	12.31	7.04	1.31E-07
naphtho[1,2-b]fluoranthene	111189-32-3	1.67	1.67	302.36	12.77	**7.28**	7.91E-08
naphtho[2,3-j]fluoranthene	205-83-4	1.67	1.67	302.36	12.77	**7.28**	7.91E-08
dibenzo[a,e]fluoranthene	5385-75-1	0.47	2.36	302.27	12.77	**7.28**	7.91E-08
dibenzo[a,l]pyrene	191-30-0	0.48	2.41	302.27	13.2	7.71	7.91E-08
naphtho[2,3-k]fluoranthene	207-18-1	1.67	1.67	302.37	12.77	**7.28**	7.91E-08
naphtho[2,3-e]pyrene	193-09-9	1.67	1.67	302.37	12.77	**7.28**	7.91E-08
dibenzo[a,e]pyrene	192-65-4	6.44	32.22	302.37	13.20	7.71	7.91E-08
coronene	191-07-1	0.70	3.49	300.35	13.70	7.64	2.12E-08
dibenzo[e,l]pyrene	192-51-8	1.67	1.67	302.36	12.77	**7.28**	7.91E-08
naphtho[2,3-a]pyrene	196-42-9	1.67	1.67	302.36	12.77	**7.28**	7.91E-08
benzo[b]perylene	197-70-6	1.67	1.67	302.36	12.77	**7.28**	7.91E-08
dibenzo[a,i]pyrene	189-55-9	1.42	7.10	302.37	12.77	**7.28**	7.91E-08
dibenzo[a,h]pyrene	189-64-0	0.52	2.60	302.37	12.77	**7.28**	7.91E-08

LOD – limit of detection; LOQ – limit of quantification

1 KOAWIN v1.10.1 predicted values [1]

2 Bold values are KOWWIN v1.67, others are experimental values [1]

3 Estimated Henry's law constant using bond method at 298 [1]

Table S2. PAH concentrations (ng/m^3) for each site and mean and standard deviation [2].

Chemical Name	Sample #1	Sample #2	Sample #3	Sample #4	Sample #5	Mean	Standard Deviation
naphthalene	2.05E+02	6.00E+01	1.88E+02	2.35E+02	2.42E+02	1.86E+02	7.4E+01
2-methylnaphthalene	9.39E+01	1.30E+01	6.80E+01	8.31E+01	8.83E+01	6.93E+01	3.3E+01
1-methylnaphthalene	6.74E+01	8.92E+00	4.19E+01	4.92E+01	5.24E+01	4.40E+01	2.2E+01
2-ethylnaphthalene	3.67E+01	4.69E+00	1.24E+01	1.44E+01	1.52E+01	1.67E+01	1.2E+01
1,2-dimethylnaphthalene	9.22E+00	1.19E+00	2.86E+00	3.17E+00	3.31E+00	3.95E+00	3.1E+00
1,4-dimethylnaphthalene	7.00E+00	8.06E-01	1.94E+00	2.12E+00	2.20E+00	2.81E+00	2.4E+00
1,5-dimethylnaphthalene	5.82E+00	6.80E-01	1.68E+00	1.82E+00	1.98E+00	2.40E+00	2.0E+00
2,6-dimethylnaphthalene	8.39E+00	1.07E+00	3.61E+00	4.21E+00	4.62E+00	4.38E+00	2.6E+00
fluorene	6.48E+01	6.35E+00	1.70E+01	1.82E+01	1.97E+01	2.52E+01	2.3E+01
phenanthrene	4.41E+01	4.00E+00	7.41E+00	7.54E+00	8.17E+00	1.42E+01	1.7E+01
dibenzothiophene	1.37E+00	1.17E-01	2.24E-01	2.20E-01	2.45E-01	4.35E-01	5.2E-01
1-methylphenanthrene	7.77E-01	5.00E-02	7.68E-02	7.60E-02	8.04E-02	2.12E-01	3.2E-01
2-methylanthracene	2.07E-01	2.58E-04	2.02E-02	1.97E-02	1.95E-02	5.33E-02	8.6E-02
2-methylphenanthrene	2.50E+00	1.78E-01	2.96E-01	3.00E-01	3.12E-01	7.17E-01	1.0E+00
9-methylanthracene	5.80E-03	3.93E-04	7.50E-04	3.93E-04	3.93E-04	1.55E-03	2.4E-03
pyrene	8.26E-01	1.64E-02	3.77E-02	3.29E-02	4.12E-02	1.91E-01	3.6E-01
fluoranthene	2.04E+00	2.95E-02	6.07E-02	5.42E-02	6.62E-02	4.50E-01	8.9E-01
2,3-dimethylanthracene	2.23E-02	5.61E-05	5.64E-05	5.60E-05	5.65E-05	4.51E-03	9.9E-03
3,6-dimethylphenanthrene	5.73E-02	2.38E-03	3.62E-03	3.67E-03	3.81E-03	1.42E-02	2.4E-02
benzo[a]fluorene	3.21E-02	2.89E-04	1.08E-03	8.46E-04	1.11E-03	7.09E-03	1.4E-02
benzo[b]fluorene	1.34E-02	9.66E-05	3.47E-04	2.96E-04	3.66E-04	2.90E-03	5.9E-03
benzo[c]fluorene	1.19E-02	1.88E-04	5.78E-04	5.02E-04	5.93E-04	2.75E-03	5.1E-03
1-methylpyrene	9.33E-03	1.52E-04	3.00E-04	2.35E-04	3.14E-04	2.07E-03	4.1E-03
chrysene	1.15E-02	1.59E-04	2.94E-04	2.19E-04	3.62E-04	2.51E-03	5.0E-03
benz[a]anthracene	1.36E-02	1.09E-04	2.96E-04	2.41E-04	3.44E-04	2.92E-03	6.0E-03
triphenylene	3.66E-03	9.71E-05	1.22E-04	9.69E-05	1.45E-04	8.24E-04	1.6E-03
retene	6.29E-02	5.21E-03	3.52E-03	3.10E-03	4.02E-03	1.58E-02	2.6E-02
benzo[b]fluoranthene	1.42E-03	5.66E-05	6.70E-05	4.86E-05	1.02E-04	3.39E-04	6.0E-04
benzo[e]pyrene	5.07E-04	2.51E-05	3.05E-05	1.82E-05	4.46E-05	1.25E-04	2.1E-04
benzo[j]fluoranthene	8.66E-04	3.53E-05	4.22E-05	3.10E-05	7.00E-05	2.09E-04	3.7E-04
benzo[k]fluoranthene	4.75E-04	7.08E-06	7.88E-06	6.65E-06	8.07E-06	1.01E-04	2.1E-04
benzo[ghi]perylene	3.10E-04	2.43E-05	2.06E-05	1.51E-05	2.94E-05	7.99E-05	1.3E-04

Table S3. List of chemicals used to create sufficiently similar mixtures, purity, and supplier.

Chemical Name	Purity	Supplier
naphthalene	99%	Sigma Aldrich
acenaphthene	100%	AccuStandard
2-methylnaphthalene	98.4%	AccuStandard
1-methylnaphthalene	99.6%	AccuStandard
fluorene	98.1%	AccuStandard
2-ethylnaphthalene	>99%	Sigma Aldrich
phenanthrene	99.5%	AccuStandard
2,6-dimethylnaphthalene	99.6%	AccuStandard
1,2-dimethylnaphthalene	94%	AccuStandard
1,4-dimethylnaphthalene	95.7%	AccuStandard
1,5-dimethylnaphthalene	100%	AccuStandard
2-methylphenanthrene	99.5%	ChemService
fluoranthene	97.2%	AccuStandard
dibenzothiophene	>99%	Sigma Aldrich
1-methylphenanthrene	99.2 +/- 0.05 %	Agilent
pyrene	98.5%	Sigma Aldrich
2-methylanthracene	100%	AccuStandard
retene	97%	Santa Cruz Biotechnology
3,6-dimethylphenanthrene	99.3%	AccuStandard
benzo[a]fluorene	>98%	Santa Cruz Biotechnology
benz[a]anthracene	100%	AccuStandard
benzo[b]fluorene	98.1%	AccuStandard
benzo[c]fluorene	98%	TRC
chrysene	99.8%	AccuStandard
1-methylpyrene	98.9%	Sigma Aldrich
triphenylene	99.4%	AccuStandard
benzo(b)fluoranthene	100%	AccuStandard
benzo(j)fluoranthene	98.1%	AccuStandard
benzo(e)pyrene	99.9%	AccuStandard
benzo(ghi)perylene	98.9%	AccuStandard

Table S4. Key to teratogenic super endpoint abbreviations with definitions

ABBREVIATION	ENDPOINT	TIMEPOINT
**MO24**	Mortality at 24 hpf	24
**SM24**	Spontaneous movement	24
**MORT**	Dies between 24 and 120 hours post fertilization (hpf)	120
**AXIS**	Curved or bent axis in either direction	120
**BRN**	Brain malformations or necrosis	120
**CRAN**	Malformed, missing or smaller than normal the eye, snout, and/or jaw	120
**EDEM**	Heart and/or yolk sac malformation, pericardial or yolk sac edema (fluid around the heart)	120
**LTRK**	Malformation of the lower trunk, including caudal fin region	120
**MUSC**	Lack of circulation, malformation or disorganized/ missing somites, and improper swim bladder formation	120
**NC**	Notochord distortion	120
**PIG**	Lack of pigmentation, overpigmentation	120
**TR**	Not responsive to touch at 120 hpf	120
**SIDE**	Upright or laying on its side	120

Table S5 Incidence (%) of effect for 13 zebrafish morphological endpoints for Abundance Mix (Abun. Mix), Creosote-Fire Mix (SC Mix), Toxicity Mix (Tox Mix) and Weighted Toxicity Mix (WA-Tox Mix) and for A.) 0- 75 µM and B.) 0-600 µM test concentrations (conc). Abbreviations for each morphological endpoint can be found in Table S4.

A.

Mixture Name	conc (uM)	MO24	DP24	SM24	MORT	CRAN	AXIS	EDEM	MUSC	LTRK	BRN_	SKIN	NC__	TCHR	any.effect
Abundance Mixture	0	2.78	0	0	5.56	5.88	2.94	2.94	0	0	0	0	2.94	0	13.89
Abundance Mixture	0.5	0	0	0	0	0	0	0	0	0	0	0	0	0	0
Abundance Mixture	1	2.78	0	0	5.56	5.88	0	5.88	0	0	0	0	0	0	11.11
Abundance Mixture	5	2.78	0	0	2.78	0	2.86	0	0	0	0	0	0	0	5.56
Abundance Mixture	10	0	0	0	0	5.56	0	2.78	0	0	0	0	0	0	5.56
Abundance Mixture	25	0	0	0	2.78	5.71	2.86	0	0	0	0	0	0	0	8.33
Abundance Mixture	50	2.78	0	0	5.56	5.88	2.94	2.94	0	0	0	0	0	0	11.11
Abundance Mixture	75	0	0	0	2.78	0	0	0	0	0	0	0	0	2.86	5.56
Creosote-Fire Mixture	0	2.78	0	0	5.56	5.88	5.88	2.94	0	0	0	0	0	2.94	11.11
Creosote-Fire Mixture	0.5	0	0	0	0	5.56	0	2.78	0	0	0	0	0	0	5.56
Creosote-Fire Mixture	1	2.78	0	0	2.78	2.86	0	0	0	0	0	0	0	0	5.56
Creosote-Fire Mixture	5	0	0	0	2.78	5.71	0	2.86	0	0	0	0	0	0	11.11
Creosote-Fire Mixture	10	2.78	0	0	2.78	5.71	0	2.86	0	2.86	0	0	0	2.86	8.33
Creosote-Fire Mixture	25	0	0	0	0	2.78	0	2.78	0	0	0	0	0	2.78	8.33
Creosote-Fire Mixture	50	2.78	0	0	2.78	5.71	0	0	0	0	0	0	0	0	8.33
Creosote-Fire Mixture	75	0	0	0	0	5.71	5.71	2.86	0	0	0	0	0	0	11.43
Toxicity Mixture	0	0	0	0	0	5.56	2.78	5.56	0	0	0	0	0	0	8.33
Toxicity Mixture	0.5	0	0	0	0	5.56	0	0	0	0	0	0	0	0	5.56
Toxicity Mixture	1	5.71	0	0	5.71	3.03	3.03	6.06	0	0	0	0	0	0	11.43
Toxicity Mixture	5	2.78	0	0	2.78	5.71	0	5.71	0	0	0	0	0	2.86	8.33
Toxicity Mixture	10	2.78	0	0	2.78	25.71	2.86	20	0	0	0	0	0	0	27.78
Toxicity Mixture	25	2.78	0	0	8.33	93.94	0	87.88	3.03	6.06	6.06	0	0	9.09	97.22
Toxicity Mixture	50	8.33	0	0	77.78	87.5	37.5	100	0	0	50	0	0	37.5	100
Toxicity Mixture	75	2.78	0	0	100	NA	NA	NA	NA	NA	NA	NA	NA	NA	100
Weighted-Toxicity Mixture	0	4.17	0	0	4.17	0	0	0	0	0	0	0	0	0	4.17
Weighted-Toxicity Mixture	0.5	0	0	0	0	8.33	4.17	4.17	4.17	4.17	0	0	0	0	8.33
Weighted-Toxicity Mixture	1	0	0	0	0	12.5	0	4.17	0	0	0	0	0	0	12.5
Weighted-Toxicity Mixture	5	0	0	0	0	0	0	0	0	0	0	0	0	0	0
Weighted-Toxicity Mixture	10	0	0	0	0	4.17	8.33	4.17	0	4.17	0	0	0	0	12.5
Weighted-Toxicity Mixture	25	0	0	0	0	0	0	0	0	0	0	0	0	0	0
Weighted-Toxicity Mixture	50	0	0	0	0	0	0	0	0	0	0	0	4.17	0	4.17
Weighted-Toxicity Mixture	75	4.17	0	0	4.17	8.7	0	4.35	4.35	0	4.35	0	0	0	12.5

B.

Mixture Name	conc (uM)	MO24	DP24	SM24	MORT	CRAN	AXIS	EDEM	MUSC	LTRK	BRN_	SKIN	NC__	TCHR	any.effect
Creosote-Fire Mixture	0	9.375	0	0	9.375	3.125	0	3.125	0	0	0	0	0	0	12.5
Creosote-Fire Mixture	200	6.25	0	0	9.375	0	0	0	0	3.125	0	0	0	0	12.5
Creosote-Fire Mixture	300	3.125	0	0	12.5	6.25	3.125	6.25	0	3.125	0	0	0	0	18.75
Creosote-Fire Mixture	400	0	0	0	6.25	3.125	0	3.125	0	0	0	0	0	0	9.375
Creosote-Fire Mixture	500	0	3.125	0	9.375	0	0	0	0	0	0	0	0	0	9.375
Creosote-Fire Mixture	600	21.875	0	0	21.875	0	0	0	0	0	0	0	0	0	21.875
Weighted-Toxicity Mixture	0	3.125	0	0	6.25	3.125	6.25	3.125	3.125	3.125	3.125	3.125	3.125	3.125	12.5
Weighted-Toxicity Mixture	200	6.25	3.125	0	12.5	3.125	3.125	3.125	3.125	6.25	3.125	3.125	3.125	3.125	18.75
Weighted-Toxicity Mixture	300	18.75	0	0	21.875	6.25	6.25	6.25	3.125	6.25	3.125	3.125	3.125	0	28.125
Weighted-Toxicity Mixture	400	3.125	3.125	0	9.375	0	3.125	3.125	0	3.125	0	0	0	0	15.625
Weighted-Toxicity Mixture	500	6.25	3.125	0	12.5	0	0	0	0	0	0	0	0	0	12.5
Weighted-Toxicity Mixture	600	9.375	9.375	0	18.75	3.125	3.125	3.125	3.125	6.25	3.125	3.125	3.125	3.125	25
Abundance Mixture	0	3.125	0	0	3.125	0	0	0	0	0	0	0	0	0	3.125
Abundance Mixture	200	6.25	0	0	9.375	0	0	0	0	0	0	0	0	0	9.375
Abundance Mixture	300	0	0	0	6.25	0	0	0	0	0	0	0	0	0	6.25
Abundance Mixture	400	0	0	0	6.25	6.25	3.125	6.25	0	0	0	0	0	0	15.625
Abundance Mixture	500	0	0	0	0	0	0	0	0	0	0	0	0	0	0
Abundance Mixture	600	21.875	0	0	21.875	3.125	0	3.125	0	0	0	0	0	0	25

Table S6. List of collected toxicity metrics for the detected PAHs in the environmental sample and associated sources for each toxicity metric. Empirically derived values are in **bold** for Cancer Potency Value (CPV), Inhalation Unit Risk (IUR), Reference Concentration (RfC), Oral Slope Factor (OSF), and Reference Dose (RfD).

PAH	RPF[3–5]	IARC Class [6]	TEF[7]	CPV (mg/kg-day)[3,5]	IUR(mg/m3)[4,5,8]	RfC(mg/m3)[3–5]	OSF(mg/kg-day)[4,5,8]	RfD (mg/kg-day)[3–5]	Zebrafish BMC50 (uM)[9]
1,2-dimethylnaphthalene			0.001	2.31	2.06E-04	9.70E-04	5.92E-01	8.28E-03	
1,4-dimethylnaphthalene			0.001	2.31	2.11E-04	9.74E-04	5.68E-01	8.23E-03	213.596
1,5-dimethylnaphthalene			0.001	2.3	2.10E-04	9.71E-04	5.76E-01	8.21E-03	
1-methylnaphthalene			0.001	2.4	1.36E-04	**0.014**	**0.029**	**0.007**	
1-methylphenanthrene		3	0.001	11.7	8.45E-04	7.02E-05	3.14E+00	3.23E-03	112.32
1-methylpyrene			0.001	37.7	1.19E-03	5.18E-05	2.69E+01	1.53E-03	413.37
2,6-dimethylnaphthalene			0.001	2	1.73E-04	9.60E-04	4.66E-01	8.48E-03	
2-ethylnaphthalene			0.001	2.27	2.03E-04	5.87E-04	2.94E-01	1.23E-02	
2-methylanthracene			0.01	11.5	5.61E-04	6.56E-05	1.99E+00	4.49E-03	
2-methylnaphthalene			0.001	2.3	1.36E-04	**0.0033**	2.83E-01	**0.004**	
2-methylphenanthrene			0.001	11.5	6.37E-04	7.04E-05	2.28E+00	4.39E-03	
3,6-dimethylphenanthrene			0.001	18.2	1.22E-03	7.00E-05	6.28E+00	3.27E-03	91.676
acenaphthene		3	0.001	4.11	3.08E-04	6.32E-04	6.16E-01	**6.00E-02**	
benz[a]anthracene	0.20	2B	0.1	**21**	**1.10E-07**	3.21E-05	**7.00E-01**	1.42E-03	59.294
benzo[a]fluorene		3	0.001	26.7	1.58E-03	3.46E-05	2.74E+01	2.36E-03	
benzo[b]fluoranthene	0.80	2B	0.1	**7.3**	**1.10E-07**	4.70E-05	**3.90E-01**	1.52E-03	
benzo[b]fluorene		3	0.001	21.4	1.86E-03	3.58E-05	4.11E+01	2.48E-03	45.869
benzo[c]fluorene	20.0	3	0.001	25.9	1.45E-03	3.61E-05	2.54E+01	2.02E-03	302.26
benzo[e]pyrene		3	1	40.4	1.93E-03	5.04E-05	2.52E+01	1.46E-03	

benzo[ghi]perylene	0.01	3	0.01	69.2	4.66E-03	5.49E-05	2.92E+01	**6.00E-02**	
benzo[j]fluoranthene	0.30	2B	0.1	**6.1**	**1.10E-03**	5.06E-05	**1.20E+00**	1.43E-03	14.483
chrysene	0.10	2B	0.01	**2**	**6.00E-10**	3.22E-05	**1.00E-03**	1.44E-03	71.85
dibenzothiophene		3	0.001	5.13	6.77E-04	2.22E-04	1.06E+00	**1.00E-02**	52.093
fluoranthene	0.08	3	0.001	25.3	1.61E-03	3.61E-05	1.50E+01	**4.00E-02**	93.646
fluorene		3	0.001	4.34	4.20E-04	4.30E-04	7.35E-01	**4.00E-02**	
naphthalene		2B	0.001	**0.12**	**2.00E-02**	**3.00E-03**	**3.40E-08**	**2.00E-02**	
phenanthrene		3	0.001	5.89	3.91E-04	5.66E-05	8.28E-01	**0.3**	105.478
pyrene		3	0.001	24.6	1.59E-03	**3.00E-02**	1.35E+01	**3.00E-02**	46.13
retene			0.001	29.7	3.09E-03	6.55E-05	3.07E+01	2.76E-03	10.426
triphenylene		3	0.001	34.2	1.21E-03	3.46E-05	2.39E+01	1.44E-03	

Table S7. Rankings of chemicals for individual toxicity metrics for Toxicity Mixture

PAH	Chemical Abundance	RfC	RfD	IUR	CPV	OSF	Zebrafish BMC	RPF
1,2-dimethylnaphthalene	8	23	20	21	25	23		
1,4-dimethylnaphthalene	10	25	19	19	24	25	12	
1,5-dimethylnaphthalene	11	24	18	20	26	24		
1-methylnaphthalene	4	26	17	25	23	30		
1-methylphenanthrene	16	16	12	12	13	15	11	
1-methylpyrene	25	10	7	11	3	4	14	
2,6-dimethylnaphthalene	9	22	21	23	29	26		
2-ethylnaphthalene	7	20	23	22	28	27		
2-methylanthracene	17	14	16	15	16	18		
2-methylnaphthalene*	3	28	14	24	27	28		
2-methylphenanthrene	12	17	15	14	15	16		
3,6-dimethylphenanthrene	19	15	13	9	12	13	8	
acenaphthene	2	21	29	18	22	22		
benz[a]anthracene	22	1	1	26	11	9	6	4
benzo[a]fluorene	20	3	9	7	6	5		
benzo[b]fluoranthene	27	7	6	28	17	12		2
benzo[b]fluorene	21	5	10	4	10	1	3	
benzo[c]fluorene	23	6	8	8	7	6	13	1
benzo[e]pyrene	29	8	5	3	2	7		

benzo[ghi]perylene	30	11	26	1	1	3		7
benzo[j]fluoranthene	28	9	4	27	18	14	2	3
chrysene	24	2	2	30	30	17	7	5
dibenzothiophene*	14	18	22	13	20	19	5	
fluoranthene*	13	30	28	5	8	10	9	6
fluorene	5	19	27	16	21	21		
naphthalene	1	27	24	29		29		
phenanthrene	6	12	30	17	19	20	10	
pyrene	15	29	25	6	9	11	4	
retene	18	13	11	2	5	2	1	
triphenylene	26	4	3	10	4	8		

Table S8. Ranking of chemicals for individual toxicity metrics for Weighted-Toxicity Mixture

PAH	IARC Class	TEF	IUR	RfC	OSF	RfD	Zfish BMC
1,2-dimethylnaphthalene		9	8	22	8	22	
1,4-dimethylnaphthalene		10	11	21	11	21	14
1,5-dimethylnaphthalene		11	12	20	12	20	
1-methylnaphthalene		4	5	30	13	26	
1-methylphenanthrene	12	17	16	15	14	15	12
1-methylpyrene		28	23	10	22	5	7
2,6-dimethylnaphthalene		8	9	23	9	23	
2-ethylnaphthalene		6	7	25	6	24	
2-methylanthracene		13	18	14	19	14	
2-methylnaphthalene		3	4	28	2	25	
2-methylphenanthrene		12	13	17	10	16	
3,6-dimethylphenanthrene		24	19	12	20	13	8
acenaphthene	17	2	2	27	1	30	
benz[a]anthracene	7	16	28	7	26	7	4
benzo[a]fluorene	10	25	20	11	17	11	
benzo[b]fluoranthene	4	20	29	4	28	3	10
benzo[b]fluorene	9	26	21	9	18	10	2
benzo[c]fluorene	8	27	22	8	21	9	6
benzo[e]pyrene	2	19	26	2	24	1	
benzo[ghi]perylene	1	30	25	1	25	8	
benzo[j]fluoranthene	3	22	27	3	27	2	1
chrysene	6	21	30	6	30	6	5
dibenzothiophene	13	15	15	18	16	17	11
fluoranthene	14	14	10	16	5	19	13
fluorene	16	5	3	26	3	27	

naphthalene	18	1	1	29	29	28	
phenanthrene	15	7	6	19	4	29	15
pyrene	11	18	14	24	7	18	9
retene		23	17	13	15	12	3
triphenylene	5	29	24	5	23	4	

Table S9. Percentage of chemicals in each sufficiently similar mixture.

**Abundance Mixture**		**Toxicity Mixture**		**Weighted-Toxicity Mixture**	
Chemical Name	Percent in Mixture	Chemical Name	Percent in Mixture	Chemical Name	Percent in Mixture
Naphthalene	44.0%	retene	72.8%	acenaphthene	48.4%
Acenaphthene	18.3%	benzo[b]fluorene	14.6%	2-methylnaphthalene	37.4%
2-methylnaphthalene	15.9%	triphenylene	7.28%	fluorene	13.6%
1-methylnaphthalene	11.9%	benzo[e]pyrene	3.64%	2-methylphenanthrene	0.387%
Fluorene	6.7%	benzo[a]fluorene	1.45%	fluoranthene	0.243%
2-ethylnaphthalene	X	benzo[c]fluorene	0.145%	benzo[j]fluoranthene	0.00011%
phenanthrene	3.16%	benzo[ghi]perylene	0.091%	benzo[e]pyrene	0.00007%

Table S10. Percentage of chemicals in 30 PAH mixture (Creosote-Fire Mixture)

**Chemical Name**	**Percentage in Mixture**
naphthalene	40.4%
acenaphthene	19.4%
2-methylnaphthalene	15.0%
1-methylnaphthalene	9.54%
fluorene	5.47%
2-ethylnaphthalene	3.62%
phenanthrene	3.09%
2,6-dimethylnaphthalene	0.951%
1,2-dimethylnaphthalene	0.857%
1,4-dimethylnaphthalene	0.610%
1,5-dimethylnaphthalene	0.520%
2-methylphenanthrene	0.155%
fluoranthene	0.0977%
dibenzothiophene	0.0944%
1-methylphenanthrene	0.0460%
pyrene	0.0414%

2-methylanthracene	0.0116%
retene	0.00342%
3,6-dimethylphenanthrene	0.00307%
benzo[a]fluorene	0.00154%
benz[a]anthracene	0.00063%
benzo[b]fluorene	0.00063%
benzo[c]fluorene	0.00060%
chrysene	0.00054%
1-methylpyrene	0.00045
triphenylene	0.00018
benzo[b]fluoranthene	0.00007
benzo[j]fluoranthene	0.00005
benzo[e]pyrene	0.00003
benzo[ghi]perylene	0.00002

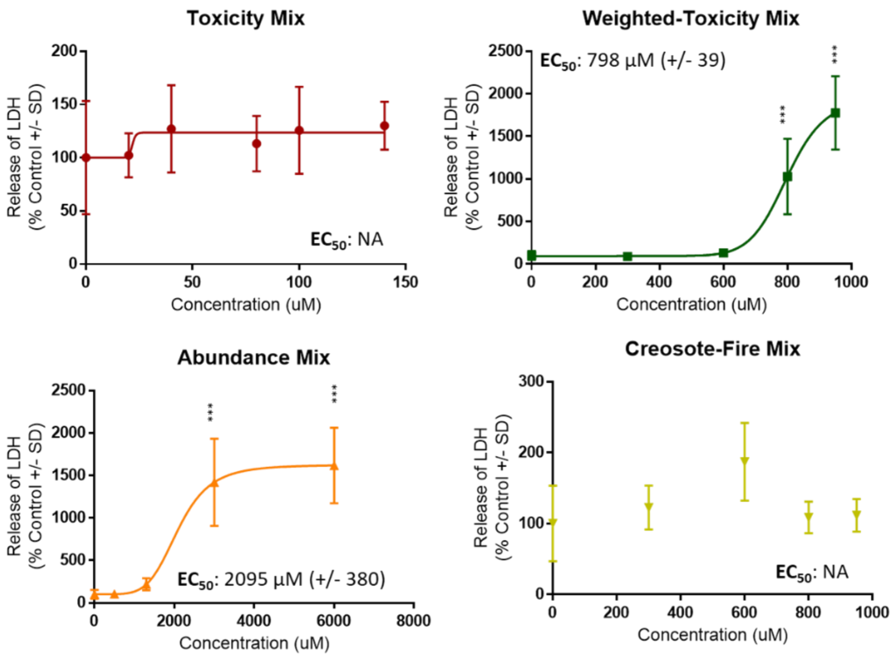

Figure S1 Concentration-Response Curves for Release of LDH in NHBE. Plots are in order of decreasing potency based on predicted EC_{50} values. Concentrations significantly different from control are denoted with an asterisk (*). $p < 0.05$ *; $p < 0.01$ **; $p < 0.001$ ***

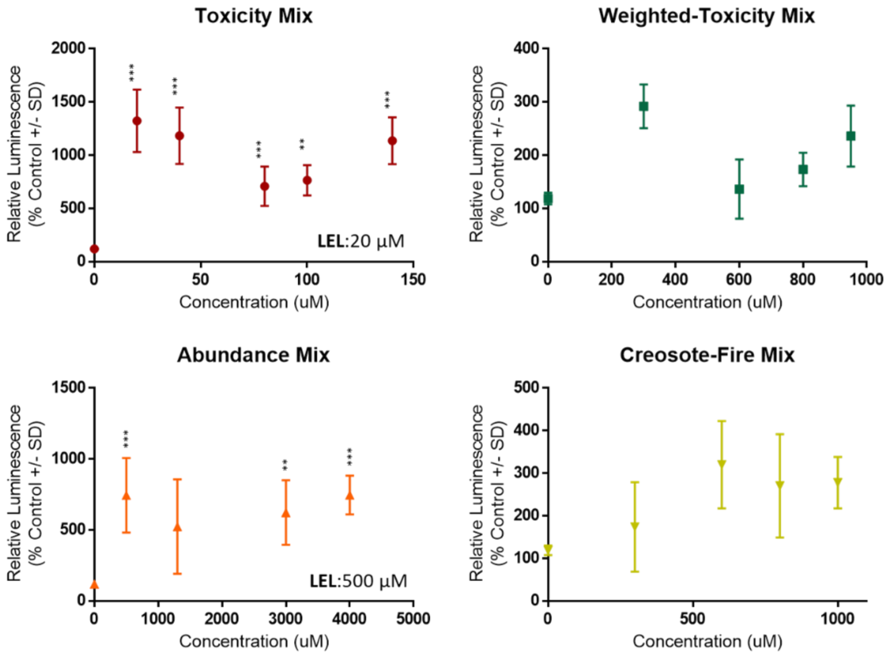

Figure S2 ROS generation in NHBE and associated lowest effect levels (LEL). LEL defined as the lowest concentration significant from control. Concentrations significantly different from control are denoted with an asterisk (*). $p < 0.05$ *; $p < 0.01$ **; $p < 0.001$ ***

Table S11. Bioassay results in 2D NHBE expressed as average percent response relative to vehicle control +/- standard deviation. Average concentrations calculated for total of n=6 from two duplicate plates for MMP and CTG. Average concentrations for total n=4 from a single plate for ROS-Glo and LDH. Concentration not tested for a given assay were denoted by NA.

Mixture Name	Concentration (µM)	Mitochondrial Membrane Potential (MMP)	Cell Viability (CTG)	Reactive Oxygen Species Generation (ROS-Glo)	Release of Lactate Dehydrogenase (LDH)
Vehicle Control	0	100 +/- 11.3%	100 +/- 14.6%	100 +/-	100 +/- 53.3%
Abundance Mixture	500	99.4 +/- 22.3%	112 +/- 11.0%	628 +/- 221%	106 +/- 27.1%
	800	83.3 +/- 24.2%	105 +/- 9.07%	NA	NA
	1300	171 +/- 49.1%	82.8 +/- 10.3%	309 +/- 121%	220 +/- 70.4%
	1600	256 +/- 52.4%	84.3 +/- 8.83%	NA	NA
	3000	328 +/- 41.4%	9.82 +/- 7.07%	441 +/- 117%	1420 +/- 511%
	4000	298 +/- 40.5%	12.9 +/- 7.01%	NA	NA

	6000	NA	NA	631 +/- 116%	1618 +/- 444%
Toxicity Mixture	20	82.4 +/- 11.9%	83.9 +/- 9.58%	1120 +/- 248%	102 +/- 20.5%
	40	298 +/- 63.6%	41.9 +/- 12.8%	NA	NA
	60	438 +/- 117 %	19.8 +/- 8.35%	NA	NA
	80	549 +/- 137%	16.4 +/- 7.83%	999 +/- 224%	127 +/- 40.9%
	100	595 +/- 56.6%	13.2 +/- 30.8%	668 +/- 85.7%	114 +/- 25.9%
	140	595 +/- 154%	14.6 +/- 3.22%	695 +/- 83.8%	126 +/- 40.8%
Weighted-Toxicity Mixture	300	104 +/- 25.8%	104 +/- 12.7%	247 +/- 34.6 %	87.5 +/- 10.1%
	600	209 +/- 39.0%	98.2 +/- 11.1%	100 +/- 48.5%	131 +/- 36.5%
	700	253 +/- 37.1%	85.6 +/- 13.3%	NA	NA
	800	272 +/- 57.7%	50.2 +/- 16.5%	146 +/- 26.5%	1030 +/- 442%
	875	263 +/- 44.4%	NA	NA	NA
	900	NA	NA	461 +/- 127%	1306 +/- 349%
	950	NA	NA	101 +/- 34.0%	1776 +/- 433%
	1000	NA	NA	199 +/- 49%	1229 +/- 399%
Creosote-Fire Mixture	300	101 +/- 15.3%	83.1 +/- 6.4%	147 +/- 88.9%	120 +/- 34.5%
	600	91.0 +/- 16.3%	89.3 +/- 8.7%	193 +/- 94.0%	188 +/- 54.9%
	700	78.8 +/- 18.7%	85.1 +/- 12.8%	NA	NA
	800	84.7 +/- 29.6%	73.9 +/- 10.9%	192 +/- 111%	109 +/- 22.5%
	875	65.0 +/- 9.4%	NA	NA	NA
	900	NA	NA	86.4 +/- 26.9%	134 +/- 49.3%
	950	NA	NA	105 +/- 25.0%	112 +/- 23.3%
	1000	NA	NA	235 +/- 51.0%	98.5 +/- 63.9%

Table S12. % Incidence of zebrafish endpoints any effect and mortality at 120 hours post fertilization (hpf). Average % incidence from 3 replicate plates with n=12 on each plate for a total n=36. Each mixture had its own set of controls for each plate.

Mixture Name	Concentration (μM)	Any effect at 120 hpf	Mortality at 120 hpf
Abundance Mixture	0	13.9 +/- 3.93%	5.53 +/- 3.93%
	0.5	0	0
	1	11.1 +/- 3.93%	5.53 +/- 3.93%
	5	5.56 +/- 7.86%	2.77 +/- 3.93%
	10	5.55 +/- 3.93%	0
	25	8.33+/- 6.81%	2.77 +/- 3.93%
	50	11.1 +/- 10.4%	5.53 +/- 3.93%
	75	5.55 +/- 3.93%	2.77 +/- 3.93%
	200	9.37 +/- 9.37%	9.37 +/- 9.37%
	300	6.25 +/- 6.25%	6.25+/- 6.25%
	400	15.6 +/- 9.37%	6.25
	500	0	0
	600	25 +/- 6.25%	21.8 +/- 9.37%
Toxicity Mixture	0	8.33 +/- 11.78%	0
	0.5	5.55 +/- 3.93%	0
	1	11.4 +/- 3.77%	5.81 +/- 4.11%
	5	8.33+/- 6.81%	2.77 +/- 3.93%
	10	27.8 +/- 10.39%	2.77 +/- 3.93%
	25	97.2 +/- 3.93%	8.33
	50	100	77.8 +/- 17.1%
	75	100	100
Weighted-Toxicity Mixture	0	4.17 +/- 4.17%	4.16 +/- 4.17%
	0.5	8.33	0
	1	12.5	0
	5	0	0
	10	12.5 +/- 4.17%	0
	25	0	0
	50	4.17 +/- 4.17%	0
	75	12.5 +/- 4.17%	4.16 +/- 4.17%
	200	18.75 +/- 12.5%	12.5 +/- 6.25%
	300	28.12 +/- 3.13 %	21.8 +/- 3.13%
	400	15.6 +/- 3.13%	9.37 +/- 3.13%
	500	12.5 +/- 6.25%	12.5 +/- 6.25%
	600	25 +/- 12.5%	18.8+/- 12.5%
Creosote-Fire Mixture	0	11.1+/- 10.4%	5.55 +/- 7.86%
	0.5	5.53+/- 3.93%	0
	1	5.53+/- 3.93%	2.77 +/- 3.93%
	5	11.1+/- 7.86%	2.77 +/- 3.93%
	10	8.33+/- 6.81%	2.77 +/- 3.93%
	25	8.33+/- 11.8%	0
	50	8.33+/- 6.81%	2.77 +/- 3.93%
	75	11.1+/- 10.4%	0
	200	12.5 +/- 6.25%	9.37 +/- 3.13%
	300	18.8 +/- 12.5%	12.5 +/- 3.93%

	400	9.37 +/- 3.13%	6.25
	500	9.37 +/- 3.13%	9.37 +/- 3.13%
	600	21.9 +/- 3.13%	21.9 +/- 3.13%

A.

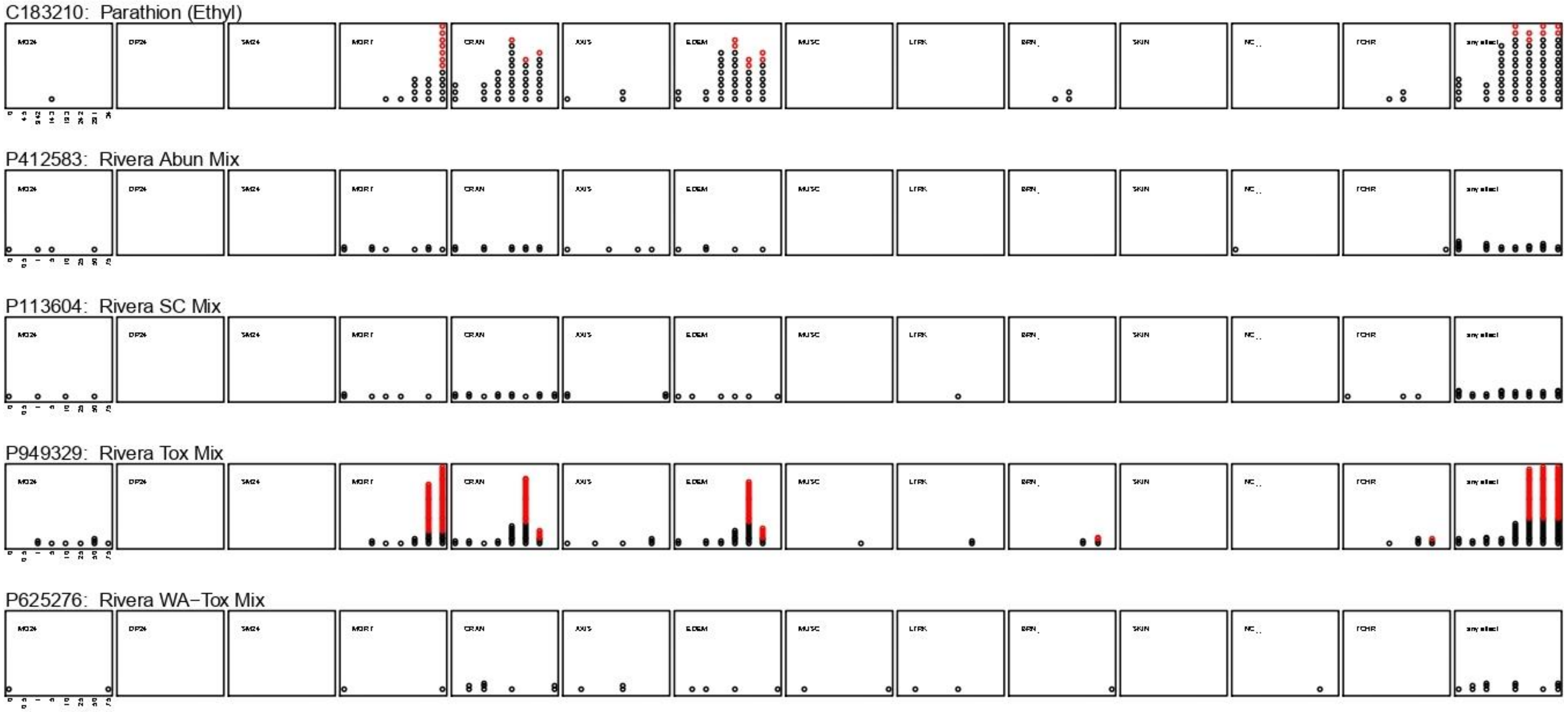
C183210: Parathion (Ethyl)
P412583: Rivera Abun Mix
P113604: Rivera SC Mix
P949329: Rivera Tox Mix
P625276: Rivera WA−Tox Mix
MO24
DP24
SM24
MORT
CRAN
AXIS
EDEM
MUSC
LTRK
SKIN
TCHR
any effect

B.

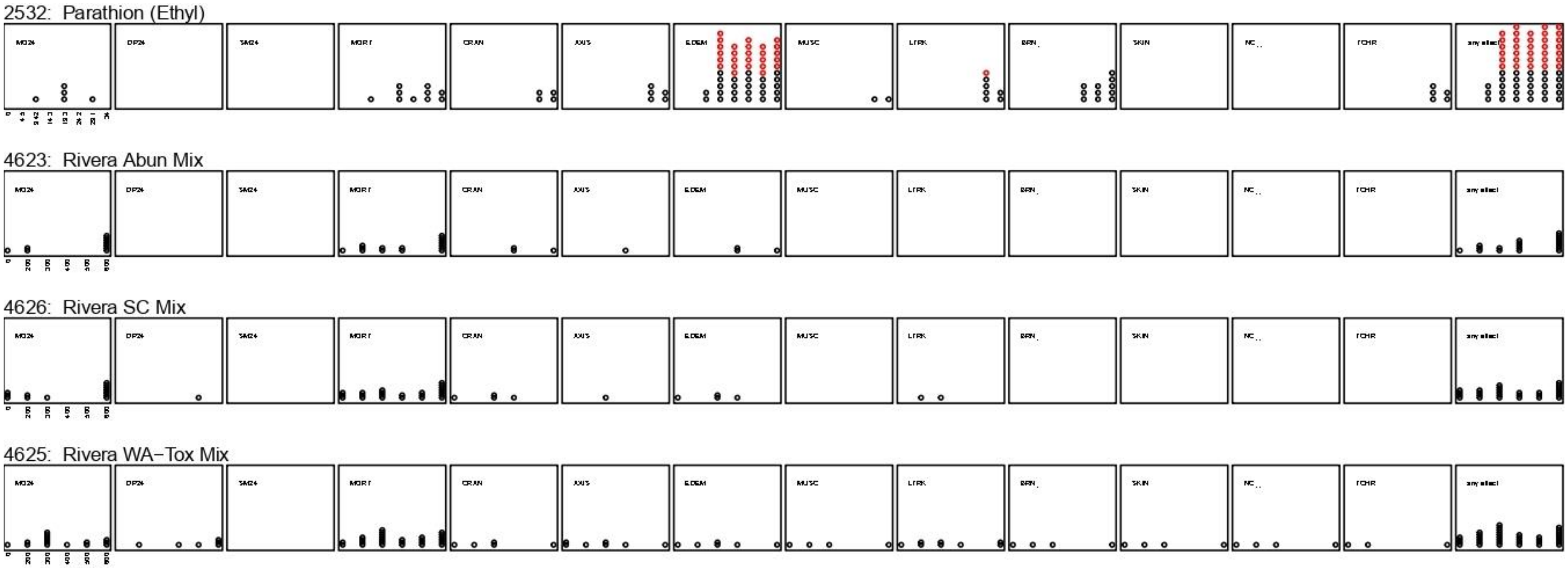

Figure S3. Concentration response plots for zebrafish morphology screening for the positive control (Parathion), Abundance Mix (Abun. Mix), Creosote-Fire Mix (SC Mix), Toxicity Mix (Tox Mix) and Weighted Toxicity Mix (WA-Tox Mix) and for A.) 0- 75 µM and B.) 0-600 µM test concentrations. Morphological effects above the binomial significance threshold are denoted with a red circle. Each red circle represents an individual hit for that morphological effect.
